# Phylogeography and Population Structure of the Invasive Land Snail *Monacha cartusiana*

**DOI:** 10.3390/ijms27104318

**Published:** 2026-05-12

**Authors:** Noreen Begum, Shumaila Noreen, Farhad Badshah, Ahmed Mahmoud Ismail, Manal Hadi Ghaffoori Kanaan, Irfan Ullah, Ahmed Othman Alsabih, Saeedah Almutairi, Aljawharah Fahad Alabbad, Mostafa A. Abdel-Maksoud, Syeda Kubra, Hamid Ur Rahman

**Affiliations:** 1 Department of Zoology, Hazara University, Mansehra, Khyber Pakhtunkhwa 21120, Pakistan; 2 Shenzhen Branch, Guangdong Laboratory of Lingnan Modern Agriculture, Key Laboratory of Livestock and Poultry Multi-Omics of MARA, Agricultural Genomics Institute at Shenzhen, Chinese Academy of Agricultural Sciences, Shenzhen 518000, China; 3 State Key Laboratory of Animal Biotech Breeding, Institute of Animal Sciences, Chinese Academy of Agricultural Sciences, Beijing 100193, China; 4 Pests and Plant Diseases Unit, College of Agricultural and Food Sciences, King Faisal University, Al-Ahsa 31982, Saudi Arabia; 5 Department of Food Industry Technologies, Technical Institute of Suwaria, Middle Technical University, Baghdad 52002, Iraq; 6 Department of Biotechnology and Genetic Engineering, Hazara University, Mansehra, Khyber Pakhtunkhwa 21120, Pakistan; 7 Department of Physiology, College of Medicine, King Saud University, Riyadh 11461, Saudi Arabia; 8 Department of Botany and Microbiology department, College of Science, King Saud University, Riyadh 11451, Saudi Arabia; 9 Research Chair of Biomedical Applications of Nanomaterials, Biochemistry Department, College of Science, King Saud University, Riyadh 11451, Saudi Arabia; 10 Aging Institute of UPMC, University of Pittsburgh School of Medicine, Pittsburgh, PA 15219, USA; syeda.ecnu@hotmail.com

**Keywords:** AMOVA, diversity, evolutionary, genetic, haplotype, pest

## Abstract

*Monacha cartusiana* (O. F. Müller, 1774), native to the Mediterranean region and Europe, is a terrestrial gastropod recognized as a highly destructive agricultural pest that causes significant damage to crop plants, fruit trees, vegetables, ornamentals, and natural ecosystems. Despite its broad geographic distribution, the evolutionary history and phylogeographic relationships of *M. cartusiana* populations remain globally unexplored. This study reports the first molecularly confirmed record of *M. cartusiana* in Pakistan and investigates its genetic diversity and phylogeographic structure within a global context using mitochondrial markers. After morphological identification, genomic DNA was extracted from collected specimens using the CTAB method, followed by amplification and sequencing of the mitochondrial *COI* and *16S rRNA* genes. The resulting sequences were subsequently analyzed using DnaSP and PopART software to estimate genetic diversity, perform neutrality tests, and construct haplotype networks. Published sequences of *M. cartusiana* retrieved from GenBank were incorporated to provide a global comparative framework. The COI dataset (555 bp) revealed 52 haplotypes, whereas the 16S rRNA dataset (269 bp) identified 14 haplotypes across global populations. High haplotype diversity (Hd = 0.946 for COI; Hd = 0.831 for 16S rRNA) and moderate nucleotide diversity (π = 0.010 for COI; π = 0.01253 for 16S rRNA) indicated substantial genetic variability within the species. Neutrality tests produced negative and insignificant values for Tajima’s D for COI and significant values for 16S rRNA (−1.428 for *COI*; −0.20586 for *16S rRNA*) and Fu’s Fs (−29.776 for COI; −1.263 for 16S rRNA), suggesting historical population expansion. Phylogenetic reconstruction and haplotype network analyses identified two major clades (Clade A and Clade B), reflecting genetic relationships among populations from different geographic regions. AMOVA based on *COI* and *16S rRNA* sequences revealed significant population structuring, with 29.98–51.30% of the total genetic variation occurring among populations and high fixation indices (FST = 0.299–0.51398, *p* = 0.001), indicating pronounced genetic differentiation and restricted gene flow. Pairwise FST analyses indicated that the Pakistani population is most closely related to populations from Italy and Central Europe, suggesting a closer genetic affinity with Southern or Central European populations. However, FST alone does not allow definitive inference of introduction directionality, and additional analyses would be required to robustly identify the source population. Overall, this study provides the first comprehensive molecular and phylogeographic assessment of the *M. cartusiana* species from Pakistan within a global context. These findings contribute important baseline data for understanding the evolutionary dynamics, dispersal history, and population connectivity of this economically important pest species. The pronounced genetic differentiation among populations and the suggested genetic affinity of the Pakistani population with European lineages have direct implications for biosecurity monitoring, invasion pathway tracing, and targeted pest management strategies. Future research integrating nuclear markers with the mitochondrial data presented here will be essential for a more complete understanding of gene flow and local adaptation in this species.

## 1. Introduction

Gastropods are fundamental to terrestrial ecosystems and hold diverse implications for human society [1]. Ecologically, they act as sensitive barometers for environmental quality; socioeconomically, they offer traditional food sources across many cultures; and epidemiologically, they act as critical intermediate hosts for a variety of parasites with severe public health and agricultural consequences [2]. Land snails have become economically significant pests, feeding on a wide range of agronomic and horticultural plants [3]. The glass clover snail, *Monacha cartusiana* (hereafter *M. cartusiana*), is widely recognized as a highly invasive species causing heavy damage to crops and vegetables [4]. Besides being an Agroecological nuisance, *M. cartusiana* acts as a mechanical carrier and a reservoir of pathogens affecting humans and livestock [5], and acts as an intermediate host for several parasites, including *Brachylaema* species, *Taenia* species, lungworm species, and *Fasciola hepatica* metacercaria [6]. It lives primarily in open grasslands, on dry, grassy slopes and in scrub, in fallow lands and meadows, but also on forest edges. It exhibits broad tolerance for varying moisture conditions [7].

Species of the genus *Monacha,* including *M. cartusiana*, originally distributed across the western Palearctic, range from Western Europe to North Africa, the Asian part of Turkey, Lebanon, the Caucasus, Iran, Arabia, Eastern Europe, Germany, France, Ukraine [8], the Czech Republic [9], Poland [10] and Iraq [11,12,13] and are characterized by distinct shell morphology: shell diameter ranges from 16 to 18 mm with yellowish coloration, and shell height from 10 to 12 mm with creamy milk-white coloration [14]. These snails are primarily nocturnal and herbivorous and constitute significant agricultural pests, adapted to a variety of host plants such as different field crops, fruit orchards, vegetables, ornamental plants, and various other plantations [15,16,17].

Increasingly, several species of mollusks on land are expanding their ranges, and alien species are emerging in the local snail fauna because of the increasing volume of global trade between different countries [18]. The global distribution of this species has been profoundly influenced by anthropochorous dispersal, the unintentional movement of organisms by human activity, which has allowed it to invade multiple continents [19]. The expansion of its range is often associated with climate change and the global trade of soil, seedlings, and agricultural products [20].

In its native European range, *M. cartusiana* is one of the most widely distributed and ecologically successful land snails. In Europe, there has been much research on its population genetics and taxonomic boundaries. Notably, Neiber and Hausdorf employed molecular phylogenetics to clarify the “cartusiana-group” and differentiate between *M. cartusiana* of Western and Central Europe and the morphologically similar *M. claustralis* of the Balkans [21]. Similarly, research by Piekowska et al. in Poland found that although the species has a relatively conserved mitochondrial profile within its native range, it has a high potential for anthropogenic range expansion [22]. These European studies are essential in providing the genetic reference necessary to determine the origin of new populations in non-native areas, including South Asia. The combination of molecular markers is crucial in overcoming the conchological homoplasy limitations in terrestrial malacology. In particular, morphological features like shell shape and the apertural rib do not reliably differentiate closely related species, e.g., *M. cartusiana* and *M. claustralis* in the genus *Monacha* [21]. The high rate of mutation of the COI gene and structural stability of the 16S rRNA gene can therefore be exploited together to construct a robust phylogenetic framework capable of correctly placing populations that would otherwise be misclassified using traditional morphological systems.

Historically, in Pakistan, the genus *Monacha* is poorly described, with no formal taxonomy. In the past, species such as *Monacha cantiana* have been documented in several local malacological surveys and checklists [23,24]. These previous records, however, were wholly based on the conchological characters, including shell size, umbilical shape, and the presence of an apertural rib. With the high level of phenotypic plasticity and the occurrence of cryptic species within the cartusiana-group, identifications entirely based on shell morphology are becoming increasingly regarded as inadequate and subject to error [25]. No research has so far been conducted in Pakistan to identify the local *Monacha* populations at the species level using molecular barcoding. It is therefore essential to combine molecular tools with traditional morphology to accurately document the occurrence and dispersal of these potentially invasive snails.

Conventionally, the taxonomic identity of terrestrial snails has been determined based on shell measurements and anatomy; morphological identification has proven challenging due to the presence of phenotypic plasticity [26,27].

The haplotype network is a widely used approach in molecular ecology and population genetics, used to understand evolutionary relationships, genetic variations, and population structure. Phylogenetic reconstructions are commonly used to depict evolution and haplotype variation occurring in populations and species by identifying the evolutionary relationships among them [28]. Haplotype networks represent a graphical framework for depicting genealogical relationships between closely related DNA sequences and are used to infer ancestral haplotypes, mutational pathways, and demographic events that could have occurred in the past, such as population growth or movement [29]. Techniques such as median-joining and statistical parsimony have been designed to estimate such relationships based on sequence diversity and mutational routes [30,31]. They are especially effective for intraspecific analyses, as these approaches permit visualization of the finer-scale evolutionary relationships that may be obscured in traditional phylogenetic trees [32]. Molecular sequence data of evolutionary lineages further enable phylogenetic analyses that provide a solid framework for testing divergence patterns among populations through model-based approaches [33]. Bioinformatics tools such as DnaSP enable estimation of haplotype diversity, nucleotide diversity and neutrality statistics from DNA sequence data, while PopART facilitates the construction and visualization of haplotype networks amenable to phylogeographic techniques [34].

*M. cartusiana* has not previously been reported from Pakistan, although several other species of the genus *Monacha,* including *Monacha cantiana* and *Monacha obstructa*, have been documented in the country [23,24,35,36]. Prior to this study, the occurrence of *M. cartusiana* in Pakistan, along with its molecular characterization and phylogenetic relationships within a global context, remained uninvestigated. The present study was therefore undertaken to address these knowledge gaps. By incorporating newly generated sequence data from Pakistani populations into a global phylogenetic framework, this research aims to elucidate patterns of genetic diversity and population structure, as well as to reconstruct the evolutionary history associated with the species’ expansion into South Asia.

## 2. Results

### 2.1. Ecology and Morphomolecular Identification of M. Cartusiana Using COI and 16S rRNA Markers

**Figure 1:**
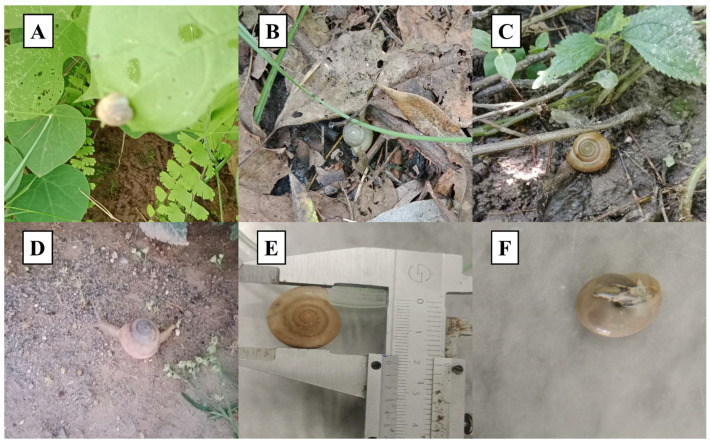
Morphological characteristics and habitat of *M. cartusiana* collected from the Hazara region, Pakistan. (**A**–**C**) Specimens observed in their natural microhabitat; (**D**) live specimen in its environment; (**E**) morphometric measurements of shell diameter using a vernier caliper and (**F**) ventral view of the shell.

*Monacha cartusiana* was most common in anthropogenic and semi-disturbed environments in Pakistan, especially in damp edges of agricultural fields, vegetable gardens and banks of irrigation canals. The snails were seen on the surface of the soil and in contact with damp leaf litter as well as aestivating on the stems of herbaceous plants (Figure 1). *M. cartusiana* was observed co-occurring with a rich gastropod fauna in these localities, most commonly the native hygromiid *Monacha obstructa*, the subulinid *Zootecus insularis*, and the genera *Bradybaena* and *Succinea*. The fact that there are juveniles and mature adults across multiple locations indicates that *M. cartusiana* has become self-sustaining in the Hazara region.

The shells of the specimens obtained (n = 380) were depressed-globose to slightly flattened-conical, with the whorls rapidly increasing (5.5 to 6.5 whorls; Figure 1), consistent with the genus *Monacha*. These shells were thin-walled, slightly translucent, of a more or less milky-white or yellowish-cream color, with a fairly smooth surface, fine growth lines, and a few slight equatorial bandings. Diagnostic characteristics were a large, widely ovate aperture with a sharp peristome strengthened internally by a clear white or reddish-brown callus, and a very narrow umbilicus covered partially by the reflected columellar margin. After morphological identification, 4 samples of the same species were evaluated through COI and 16S rRNA markers to confirm the identification of *M. cartusiana* species. Genomic DNA extraction yielded high-quality and high-concentration templates. Successful PCR amplification of the target mitochondrial markers, COI and 16S rRNA, was confirmed by the presence of distinct bands of the expected size. The amplification and sequencing success rate were 100%. After successful sequencing, alignment and trimming, the final consensus sequences for both markers were submitted to GenBank under accession numbers COI: (PX591882-PX591885) and 16S rRNA: (PX591889-PX591892). Finally, shells and the remaining tissue of the sequenced specimens were stored in 95% ethanol as permanent vouchers in the Department of Zoology at Hazara University, Mansehra, Pakistan. It is possible to use these specimens in the future as a taxonomic reference under the catalog numbers HU-Zoo-NB-2024/01-04.

### 2.2. Haplotype Network and Geographic Distribution of M. Cartusiana Based on the COI Gene

**Figure 2:**
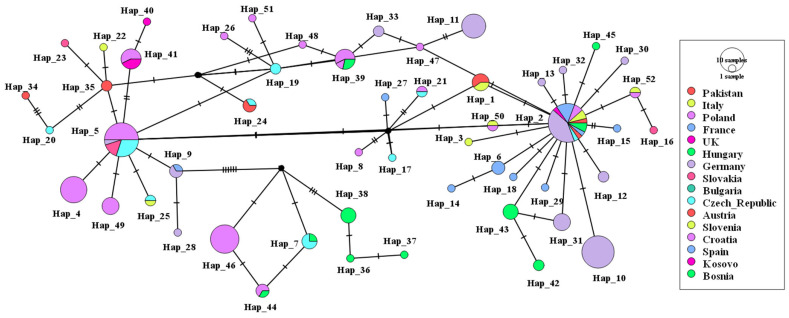
COI sequences were used to build a TCS network that comprised 189 and 52 haplotypes. With the resulting network, the size of nodes corresponding to the haplotype frequency and the connecting branches corresponding to the mutational steps, it was obvious that there was an overall topological structure. The most frequent haplotypes (Hap 2, Hap 5) hold central, star-like positions. Hatch marks on the connecting branches represent the number of mutational steps (nucleotide substitutions) separating the haplotypes. Small black circles represent inferred, unsampled median vectors (ancestral nodes). The novel sequence from Pakistan generated in this study shares haplotypes (Hap_1 and 2) with central Europe.

Analysis of 189 COI sequences (185 from GenBank with the four sequences of the current study) identified 52 distinct haplotypes (Figure 2, Table S1), revealing a complex global population structure. Haplotype distribution was highly heterogeneous. The most widespread haplotype (Hap_2, n = 26) was found across ten countries, including Pakistan. Other major haplotypes showed more restricted distributions: Hap_5 (n = 20) in Central Europe (Poland, Czech Republic, Slovakia, Germany), Hap_10 (n = 18) exclusively in Germany, and Hap_46 (n = 14) and Hap_39 (n = 7) in Poland. There were 17 haplotypes with two to five sequences, and 28 haplotypes were singletons, with each being country-specific. Figure 2 shows the statistical parsimony network of these haplotypes, and the most common (Hap 2, Hap 5) ones are the ones located in the center.

### 2.3. Haplotype Network and Geographic Distribution of M. Cartusiana Based on 16S rRNA Gene

**Figure 3:**
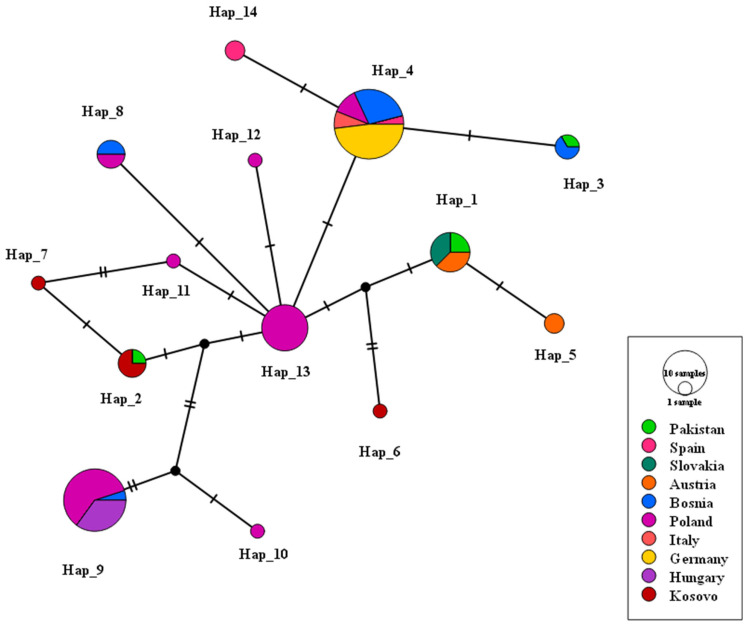
Network of 16S rRNA haplotypes inferred using statistical parsimony on 16S rRNA 84 sequences (including 16S rRNA sequences that had been created by this process), showing 14 haplotypes. The size of circles is proportional to the frequency of the haplotype, and the lines that connect are mutational steps. Hap_4 represents the most widespread and central haplotype and has a wide geographic range (Spain, Germany, Bosnia, Poland and Italy), with other haplotypes having more limited ranges. Hatch marks on the connecting branches represent the number of mutational steps (nucleotide substitutions) separating the haplotypes. Small black circles represent inferred, unsampled median vectors (ancestral nodes).

For the 16S rRNA gene, 84 sequences (including new data) yielded 14 haplotypes (Figure 3, Table S2). The most common, Hap_4 (n = 25), was geographically widespread (Spain, Germany, Bosnia, Poland, Italy). Hap_9 (n = 20) was found only in Poland, followed by Hap_13 (n = 11; Poland, Slovakia) and Hap_1 (n = 8; Pakistan, Austria). Six haplotypes were represented by 2–4 sequences, and the remaining 6 were singletons. The 16S rRNA network (Figure 3) showed a less complex topology than the COI network, with one dominant central haplotype (Hap_4).

### 2.4. Phylogenetic Analysis Based on COI Gene Haplotypes

**Figure 4:**
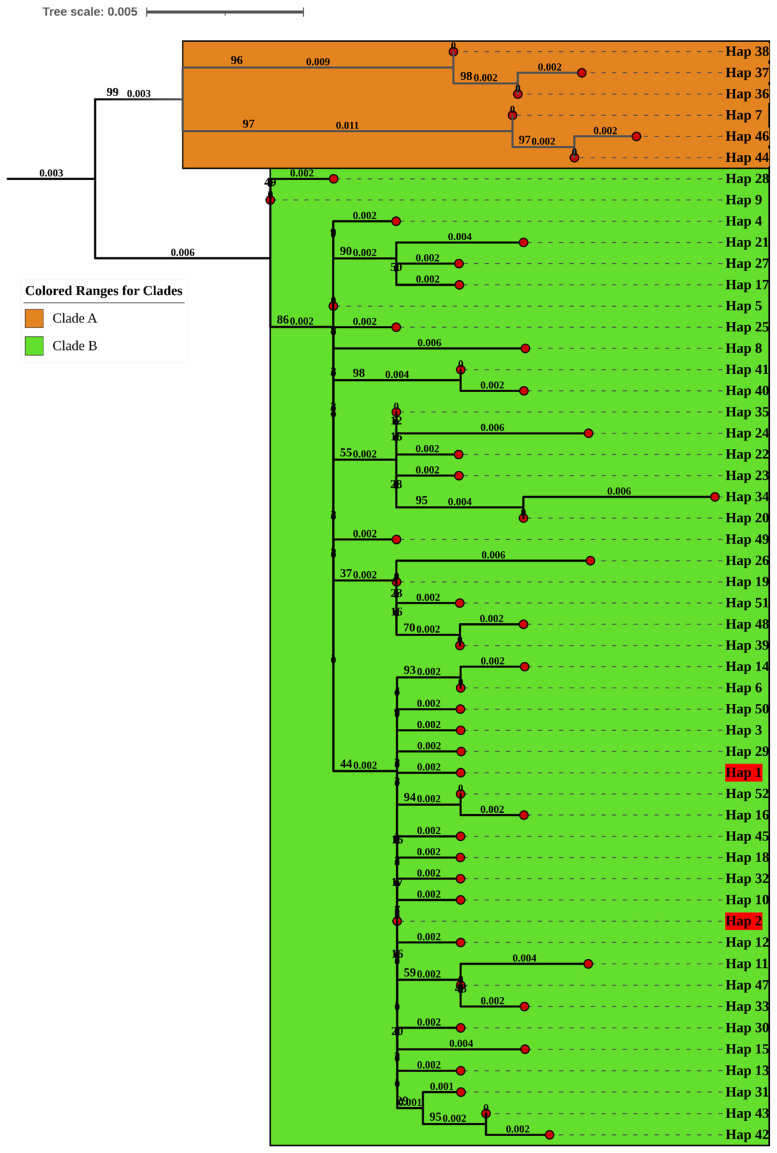
Maximum Likelihood phylogenetic tree of COI haplotypes with two large clades (A and B) with high bootstrap support; Clade B (those in high bootstrap support) has the most haplotypes and all geographic regions, including Pakistani haplotypes in red color.

The Maximum Likelihood phylogeny of COI haplotypes resolved two major clades (A and B) with strong bootstrap support (Figure 4). Clade A contained only 6 haplotypes, all from Hungary, Poland and Bosnia. Clade encompassed the majority of genetic diversity and all sampled geographic regions, including the haplotypes from Pakistan identified in this study.

### 2.5. Phylogenetic Analysis Based on 16S rRNA Gene Haplotypes

**Figure 5:**
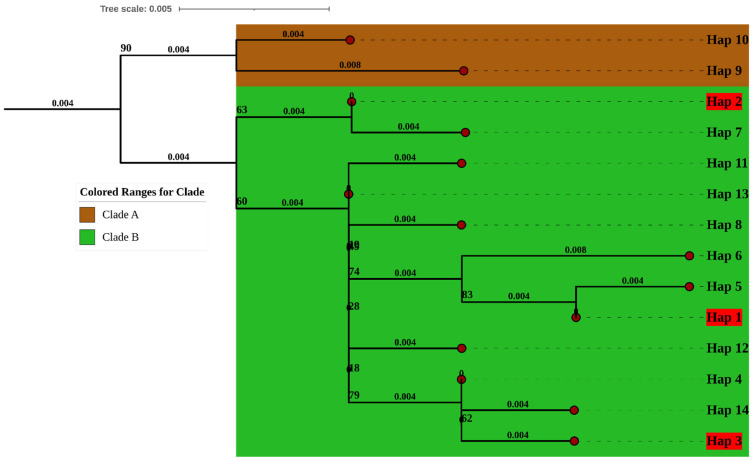
Maximum Likelihood phylogenetic tree of 16S rRNA haplotypes with two main clades (A and B), with clade B having the broadest geographic distribution in Europe and Pakistan. The haplotypes in red show the haplotypes of the current study.

The 16S rRNA haplotype phylogeny also recovered two primary clades (A and B) (Figure 5). Clade A contained just two haplotypes from Hungary, Bosnia and Poland. Clade B exhibited a broader geographic range, including sequences from Pakistan, Kosovo, Slovakia, Austria, Bosnia, Poland, Spain, Germany, and Italy.

### 2.6. Population Genetic Diversity and Neutrality Tests for the COI Gene

Analysis of 555 bp of the *COI* gene across 189 sequences revealed 53 polymorphic sites, defining 52 haplotypes. There was high haplotype diversity (Hd = 0.946) and low nucleotide diversity (0.010), defining genetic diversity (Table 1). Neutrality tests yielded significantly negative values for Fu’s Fs (−29.776, *p* < 0.001), Fu and Li’s D (−3.281, *p* < 0.02), and Fu and Li’s F (−2.890, *p* < 0.05). Tajima’s D was also negative but not significant (−1.428, *p* > 0.05).

### 2.7. Population Genetic Diversity and Neutrality Tests for 16S rRNA Gene

For the 269 bp 16S rRNA fragment (84 sequences), we found 18 polymorphic sites defining 14 haplotypes. Similar to COI, diversity indices showed high Hd (0.831) and low π (0.013) (Table 2). However, all neutrality statistics (Tajima’s D = −0.206; Fu’s Fs = −1.263; Fu and Li’s D = −0.165; Fu and Li’s F = −0.214) were negative but non-significant (*p* > 0.05). This suggests that, for this more conserved gene, the population does not show a strong signature of recent demographic change.

### 2.8. Population Differentiation and Structure (AMOVA and FST) Based on the COI Gene

**Figure 6:**
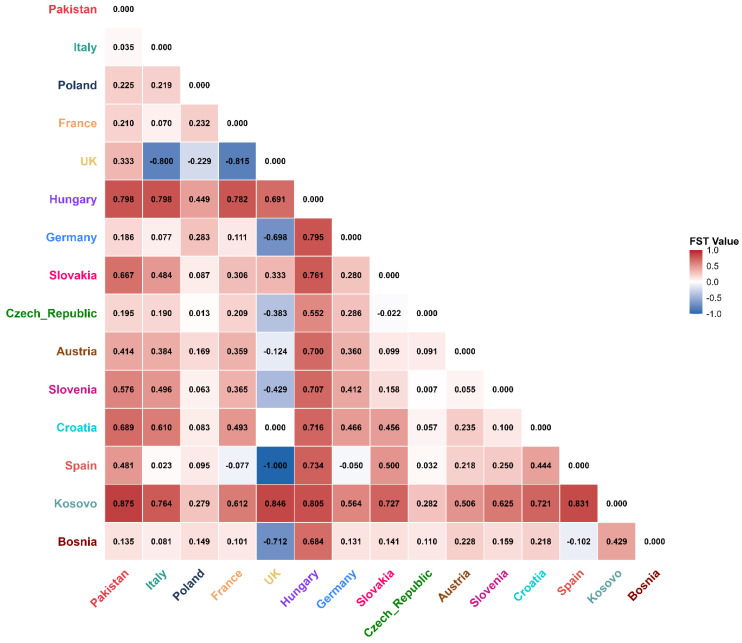
Pairwise FST values of 15 *Monacha cartusiana* populations heatmap. The color gradient is the extent of genetic differentiation, where red corresponds to larger FST values (greater differentiation) and blue/white corresponds to smaller FST values (greater genetic similarity). The population of Pakistan has the nearest genetic relationship with the Italians and Bosnian populations.

The AMOVA indicated the existence of genetic structuring, where 29.98 percent of the variance between populations and 70.02 percent within populations exists (FST = 0.299, *p* < 0.0001, based on 1023 permutations) (Table 3). The values of the pairwise FST were very diverse (Figure 6). Compared to both the Italian population (FST = 0.035) and the Bosnian population (FST = 0.135), the Pakistani population showed the lowest genetic differentiation, indicating the greatest genetic affinity with these populations among all comparisons. There was also low differentiation between the Pakistan and Germany (FST = 0.186) and the Czech Republic population (FST = 0.195). In contrast, the Pakistani population exhibited high genetic differentiation from populations in Kosovo (FST = 0.875) and Hungary (FST = 0.875). In Europe, some groups demonstrated close to zero or negative FST (e.g., Slovakia vs. Czech Republic FST = −0.022), while the populations of the UK and Spain also showed low differentiation as compared to the France and Italy groups. Negative FST values, interpreted as zero genetic differentiation, were found in a few comparisons.

### 2.9. Population Structure and Differentiation (AMOVA and FST) Based on the 16S rRNA Gene

**Figure 7:**
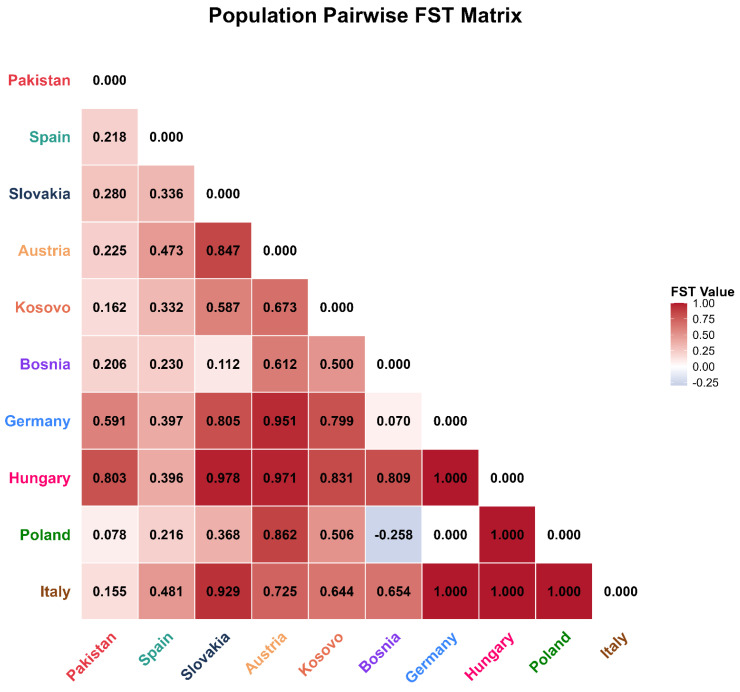
Pairwise FST estimates using 16S rRNA data in the form of a heatmap with a value of 1.000 (dark red color) indicating a high level of genetic isolation. The ones showing lower values (light/blue color) indicate a low level of genetic differentiation.

AMOVA of the 16S rRNA data indicated even stronger among-population differentiation, accounting for 51.40% of the variance (FST = 0.514, *p* < 0.001) (Table 4). Pairwise FST estimates ranged from −0.258 to 1.000 (Figure 7). Most comparisons yielded moderate to very high significant FST values, with several exceeding 0.80, pointing to pronounced genetic isolation between specific populations. The 16S rRNA data showed that the Pakistani population has the highest genetic similarity to the population from Poland (FST = 0.078), Italy (FST = 0.155) and Kosovo) FST = 0.162). The 16S marker revealed a high degree of genetic differentiation between Pakistan and Hungary (FST = 0.803) and Germany (FST = 0.591). Several European populations exhibited extremely low or negative genetic differentiation, mostly notable Bosnia vs. Poland (FST = −0.258). Conversely, the maximum differentiation value (FST = 1.000) was observed between Hungary and several other populations (Germany, Poland, Italy).

## 3. Discussion

The terrestrial snail *M. cartusiana* poses a two-fold challenge since it is considered both a common agricultural pest and a possible intermediate snail host for parasites [13]. A clear understanding of its population history is necessary to manage and understand its invasion pathways. The current study is the first genetic evidence of *M. cartusiana* from Pakistan, and this local population has been incorporated into a global phylogeographic pattern of the species through the use of COI and the 16S rRNA markers. Mitochondrial DNA is an appropriate choice for this study; its maternal transmission and lack of recombination provide a distinct single locus genealogical signal that has proved effective in gastropod phylogeographic investigation [37,38]. Although COI and 16S rRNA markers offered excellent resolution for *M. cartusiana* identification in this study, the limitations of relying solely on mitochondrial DNA (mtDNA) must be acknowledged. Mitochondrial phylogenies may occasionally be confounded by incomplete lineage sorting (ILS), in which the ancestral polymorphisms are maintained by rapid events of speciation, or by introgression due to interspecific hybridization [39]. Given the cryptic nature of the *Monacha* genus and the sympatry of the *M. cartusiana* and *M. claustralis* in their native European habitats, the possibility of mitochondrial capture by hybridization cannot be completely excluded. Although the congruence of our COI and 16S datasets confirms the accuracy of our identification, future studies incorporating nuclear markers or multi-locus genomic data would be necessary to verify these trends and provide a more comprehensive picture of the evolutionary history of *Monacha* species in South Asia.

Up to now, the presence of *M. cartusiana* has been reported extensively in Europe. It does not occur in North Africa, unlike some of the other representatives of the genus. The species is non-native in Asia and only has local introductions [21]. Its presence in Pakistan has never been established by molecular means, and the present research confirming it constitutes a major extension of its introduced range in South Asia, probably facilitated by the international trade of *M. cartusiana* [40].

Taxonomic separation of *M. cartusiana* has in the past been difficult because of the morphological conservation in the genus *Monacha*. With the extensive revision of the genus by Hausdorf, [25], many members of the *Monacha* subgenus are almost identical, depending on shell characters alone. Subsequent molecular systematic revisions by Neiber and Hausdorf (2017) and Neiber et al. (2017) have further defined the genetic identity of *M. cartusiana* [21,41].

The present study provides the first molecular description and genetic characterization of *M. cartusiana* from Pakistan, based on mitochondrial markers COI and 16S rRNA. Specimens were obtained from 21 localities across the Hazara region of Khyber Pakhtunkhwa, Pakistan (Table 5). Sequences were compared with global datasets to confirm species identity and determine phylogenetic affinity. Although *M. cartusiana* has a broad distribution in Europe, this study is the first to provide molecular confirmation of the species in South Asia (Pakistan), implying a substantial extension of its global range.

The specimens from Pakistan are morphologically consistent with *M. cartusiana*, including the narrow umbilicus and the inner rib of the aperture. Nevertheless, conchological characteristics, as noted by earlier researchers [21], do not always allow distinction between *M. cartusiana* and *M. claustralis*. The combination of shell morphology and the COI and 16S sequence data presented in this study provides a solid identification as the first confirmed record of the European lineage of *M. cartusiana* in Pakistan.

These findings provide new molecular confirmation of *M. cartusiana* in Pakistan and constitute important baseline data for examining its genetic diversity, phylogeographic distribution and dispersal history in the region. Not only does this record extend the known geographic range of the species, but it also provides a foundation for future research on population genetics, ecological impact, and management of this economically significant pest.

The results of our analysis demonstrated strong genetic differentiation between markers. We identified 52 distinct COI haplotypes and 14 of 16S rRNA haplotypes. This was expected, as the *COI* gene is more susceptible to population-level variation owing to its faster evolutionary rate. Its well-conserved function, combined with high third-codon position mutation rates, makes it a powerful marker for gastropod species identification. The number of COI haplotypes is indicative of a large diversity of maternal lineage within the species. Phylogenetic reconstruction of these haplotypes revealed a pattern typical of European fauna: one dominant, widespread clade containing the majority of samples (including Pakistani specimens) and several smaller, geographically localized clades. The nesting of Pakistani haplotypes within the major clade, rather than as a distinct early-branching lineage, strongly suggests a relatively recent introduction to South Asia, possibly associated with human demographic or dispersal movements [42]. This implies that the introduction of *M. cartusiana* into Pakistan likely occurred through recent jump-dispersal events facilitated by human activities, such as international trade or transportation of agricultural products, as has been observed in the introduction patterns of other invasive hygromiids such as *Cernuella virgata* [21,43].

Our phylogenetic analysis based on both COI and 16S rRNA haplotypes indicates that *M. cartusiana* has two general clades (groups), yet the total genetic variability is concentrated in the larger group, while the minor group contains few haplotypes. For COI, one large clade (B) covered the majority of haplotypes across many countries, while only a few were observed in the smaller clades. The 16S rRNA data similarly had a similar pattern with the main clade B containing haplotypes of many regions, and only a few of them occurred in the smaller clade A. As in the previous study, snail *Eobania vermiculata*, COI and 16S rRNA variables depicted multiple haplotypes, which belong to groups with varying geographic spreads, indicating historical occurrences of isolation and expansion in the Mediterranean area [44]. Other studies on European snails have also reported extensive COI lineages with smaller local clusters, explained by post-glacial population expansions and local isolation during climatic shifts [45]. The wide distribution of the main clade in the current study suggests that *M. cartusiana* has undergone historical gene flow and range expansion across Europe and into Pakistan, while the smaller clades may represent older or more isolated lineages that survived in restricted areas.

The population genetic statistics strongly favor a scenario of recent demographic expansion, particularly for COI. We observed the classic pattern of high haplotype diversity (Hd = 0.946) combined with moderate nucleotide diversity (π = 0.010). This “high Hd, low π” pattern typically emerges when a population grows rapidly from a small founder group, accumulating many new mutations on closely related genomes [46]. These values are comparable to those reported for *Nerita yoldii* using the *COI* gene (Hd = 0.5915, π = 0.0025) [47]. A comparative analysis of *COI* vs. *16S rRNA* in vertebrates has suggested that *COI* exhibits greater intraspecific variability, making it more sensitive to recent population change, while 16S rRNA is more conservative [48].

Neutrality tests involving *M. cartusiana* using the mitochondrial *COI* gene revealed strongly negative values, and Fu’s Fs and Fu and Li’s D and F had significant values compared to Tajima’s D, which was negative but not significant. This trend is associated with a surplus of rare haplotypes and is more likely to be attributed to rapid population growth or demographic bottleneck than to strict neutrality. The relatively large average number of pairwise nucleotide differences (K = 5.256) further confirms the accumulation of genetic variation among *COI* haplotypes. In contrast, neutrality tests for the *16S rRNA* gene produced negative but statistically non-significant values, indicating no significant departure from neutral evolution. This is explained by the lower mutation rate of 16S rRNA, which results in a reduced mean number of pairwise differences (K = 3.345). Similar trends have been noticed in recent molluscan research: COI and 16S rRNA typically indicate long-term population stability, but COI is more sensitive to recent demographic events [49]. Supporting this, Fu’s Fs returned significantly negative values for COI, rejecting a stable population model, while the 16S rRNA data were weaker, as expected for a more conserved locus [50].

AMOVA statistics reflect limited gene flow under the influence of geographic or ecological dispersal barriers. As an example, a mitogenomic study of the rice leaf folder *Cnaphalocrocis medinalis* in India and South Asia found that more than 60% *COI* variation occurred among populations, with high FST values indicating intense population isolation, probably due to reduced mobility and habitat fragmentation [51]. AMOVA of 16S rRNA data for *M. cartusiana* revealed that 51.40% of genetic diversity was distributed among populations, with a high and significant overall FST of 0.514 (*p* < 0.001), demonstrating high genetic structuring and limited gene flow. This degree of differentiation in a conservative mitochondrial marker is consistent with recent gastropod studies: 16S rDNA-based population genetic analysis of *Physella acuta* in Thailand revealed distinct population structure and evidence of genetic exchange despite limited geographic range [52].

The FST heatmap reveals that the Pakistani population exhibits the lowest genetic differentiation with populations from Southern and Central Europe, particularly Italy (FST = 0.035) and Bosnia (FST = 0.135). While this pattern is consistent with a European origin, it is important to note that FST primarily reflects levels of genetic divergence and does not directly establish the directionality of gene flow or confirm a source population. These results should therefore be interpreted as indicative of genetic affinity rather than definitive evidence of an introduction pathway. Nevertheless, this pattern is broadly consistent with human-mediated jump-dispersal, in which individuals genetically similar to a source population are transported over long distances via trade routes.

The congruence of COI and 16S FST matrices strengthens confidence in the observed pattern of genetic affinity. Both markers indicate low genetic differentiation between the Pakistani population of *M. cartusiana* and populations in Italy and Central Europe (Poland/Bosnia). The 16S data also revealed very low FST (0.078) with Poland and Italy (0.155). This concordance across two mitochondrial loci indicates a relatively recent introduction event into Pakistan, presumably via human-mediated transport, as opposed to a sustained natural range expansion. However, as FST reflects genetic divergence rather than directionality, the precise source population cannot be conclusively identified from these data alone.

The low FST values between the Pakistani and Central European populations are consistent with the findings of Pieńkowska et al., who observed that newly established populations tend to be highly similar to their source population because of rapid anthropogenic dispersal [22]. In addition, the taxonomic boundaries established by Neiber and Hausdorf, differentiating true *M. cartusiana* from the Balkan *M. claustralis* [21], confirm that the Pakistani specimens belong to the Western Palaearctic expansion lineage.

In large-scale analyses of mitochondrial diversity in insects, the non-recombining nature of the mtDNA has been highlighted as a key feature; mitochondrial variation patterns are primarily explained by demographic processes and selection rather than recombination [53]. Likewise, gastropod and other mollusk studies have demonstrated the utility of mitochondrial markers in tracking evolutionary history, as mtDNA variation reflects lineage-specific demographic processes without the confounding effect of recombination [54].

## 4. Materials and Methods

### 4.1. Study Site

**Figure 8:**
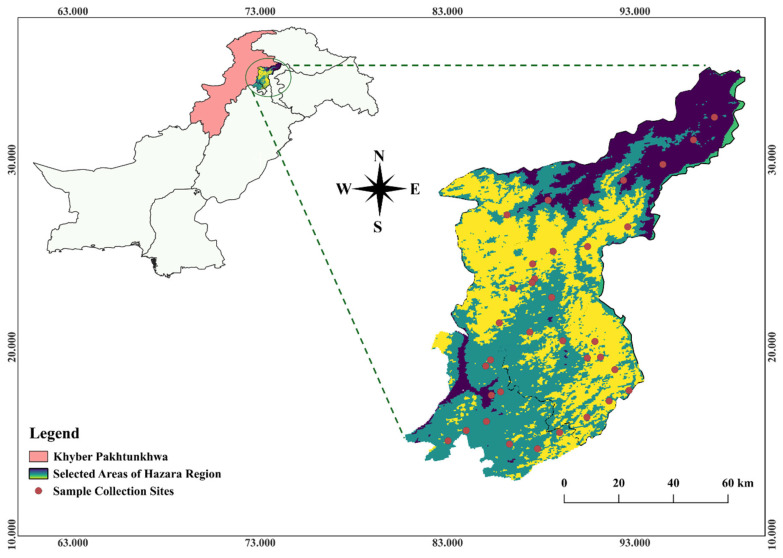
Map of the study area, showing the selected districts (Abbottabad, Mansehra, Battagram and Haripur) in the Hazara Region, Khyber Pakhtunkhwa in Pakistan.

In the current study, a total of 4600 samples of terrestrial snails were collected from four districts (Abbottabad, Mansehra, Haripur and Battagram), in the Hazara region of Pakistan (Table 5) between April 2024 and April 2025 (Figure 8) to assess the overall malacofauna. Of these, 380 specimens were morphologically identified as *M. cartusiana* based on shell morphology, while the remaining specimens consisted of other locally common taxa, including species of Bradybaenidae, Hygromiidae, Succineidae, and Zonitidae. The geographical location of the study is around 34.8344° N, 73.2253° E (Table 5), which has a bimodal rainfall distribution (February-March, and July-August) [55]. The climate of the Hazara region, combined with diverse altitudinal gradients, supports a rich gastropod fauna and makes it an ideal location for this investigation.

### 4.2. Morphological and Molecular Identification: Genomic DNA Extraction, PCR and Sequencing

Initial identification of specimens was carried out based on shell morphology using standard taxonomic keys for terrestrial gastropods. Shell characters, including shell height, shell width, aperture height, aperture width, number of whorls and umbilicus diameter, were measured using a digital vernier caliper (accuracy ±0.01 mm). Diagnostic features such as shell shape, coloration and sculpture were examined and compared with published descriptions of *M. cartusiana* [56,57] to confirm species identity.

After morphological identification, the CTAB (Cetyl Trimethyl Ammonium Bromide) method was used to extract total genomic DNA from the foot tissue of each specimen, but with some changes (longer incubation period and extra wash steps) [58]. Twenty milligrams of the foot muscles were finely crushed into powder in a sterile mortar and pestle. After crushing, 800 µL of pre-heat 2% CTAB extraction buffer (kept at 65 °C) was added, and it was vortexed vigorously. The homogenate was kept at 65 °C. Upon incubation, 600 µL of the phenol: chloroform: isoamyl alcohol (25: 24: 1, *v*/*v*/*v*) was added and centrifuged at 13,000 rpm after 20 min. The resulting supernatant was pipetted to a second tube in the presence of 500µL ice-cold isopropanol and stored overnight at −20 °C to precipitate the DNA. The samples were then centrifuged at 13,000 rpm for 20 min, and the supernatant was thrown away. Three washes of the pellet of DNA were performed using 70 percent of the ethanol before each wash by centrifuging at 13,000 rpm for 3 min. The purified DNA as a pellet was suspended in 80 µL of the double-distilled water (ddH_2_O) by air-drying of the inverted tubes. Isolated genomic DNA integrity and quality were assessed using the 1% agarose gel.

PCR was performed in 35 μL final volume to amplify two target genes (*COI* and *16S r RNA*); with the following components: 20 µL of double-distilled water (ddH_2_O), 2 µL of genomic DNA template, 3.5 µL of 10× PCR buffer, 3 µL of MgCl_2_, 2 µL of dNTPs, 0.5 units of Taq DNA polymerase, and 2 µL each of forward and reverse primers. Amplification was carried out using an Applied Biosystems 2720 Thermal Cycler with the conditions given in Table 6. The amplification was further validated by loading a 5μL aliquot of each product of the PCR into a 1.5% aliquot of agarose gel [59]. Resultant PCR amplified products were sequenced through the Sanger sequencing method from Macrogen Inc., Seoul, Republic of Korea.

### 4.3. BLAST Analysis, Compilation and Alignment of Sequences

Following sequencing, chromatograms were checked, and sequences were assembled using BioEdit version 7.2.5.0., [62]. Preliminary identification was confirmed by performing BLASTn searches against the NCBI database (https://blast.ncbi.nlm.nih.gov/Blast.cgi). After confirmation, the sequences were submitted to NCBI GenBank for accession numbers.

For global phylogeography, additional sequences of *M. cartusiana* were retrieved from GenBank for both genes, resulting in a final dataset of 189 COI and 84 16S rRNA sequences (Tables S3 and S4). Sequences with ambiguous bases were excluded. Only sequences with complete collection locality information were used for subsequent population genetic analyses and were aligned through BioEdit [62]. Retrieved sequences were aligned using the online MPI Bioinformatics Toolkit (https://toolkit.tuebingen.mpg.de/tools/mafft) utilizing MAFFT v7.515 [63], selected for its ability to incorporate secondary structure information through the Four-way Consistency objective function, which is essential for accurate alignment of non-coding ribosomal RNA. The resulting alignment file was downloaded in FASTA format, trimmed to a uniform length in BioEdit, and saved as NEXUS using MEGA 12.0.11 [64]. The final dataset consisted of 555 base pairs (bp) for *COI* and 269 bp for *16S rRNA* genes. Consequently, haplotypes in this study are defined solely across 555 bp and 269 windows.

### 4.4. Construction of Haplotype Network Using COI and 16S rRNA Sequences

Haplotype numbers for each gene were determined using DnaSP v6.12.03 [34]. The resulting haplotype data were used to construct statistical parsimony networks employing the TCS method in PopART [32] to determine relationships among *COI* and *16S rRNA* haplotypes in *M. cartusiana* populations.

### 4.5. Phylogenetic Analysis of Sequences

The phylogenetic analyses for *M. cartusiana* were performed using COI and 16S rRNA haplotype sequences. Haplotype data from DnaSP v6.12.03 were exported in FASTA format and imported into IQ-TREE version 2.2.0 [65] for Maximum Likelihood tree construction. Automated model selection was performed using ModelFinder [66], implemented in IQ-TREE version 2.2.0 with model selection based on the Bayesian Information Criterion (BIC). The TPM2u+F+I model was identified as the best-fit model for COI and F81+F for 16S rRNA. Node support was assessed using the Ultrafast Bootstrap (UFBoot2) with 1000 replicates [67]. Trees were exported in Newick format and annotated using the Interactive Tree Of Life (iTOL) v.6 web tool [68]. 

### 4.6. Neutrality Test and Population Structure Analysis

Population diversity indices [number of segregating sites (S), total number of mutations, number of haplotypes (H), haplotype diversity (Hd), nucleotide diversity (π) and average number of pairwise nucleotide differences (K)] were calculated for all datasets using DnaSP v6.12.03 [34]. Selective neutrality was tested using DnaSP by calculating Tajima’s D, Fu and Li’s D, Fu and Li’s F, and Fu’s Fs [69]. Genetic differentiation was evaluated within and among populations using Analysis of Molecular Variance (AMOVA), with populations defined by geographic boundaries. The fixation index (FST) was used to compute genetic differentiation between populations with 10,000 permutations in Arlequin version 3.5 [70]. Pairwise FST values were compared to measure the level of population divergence, with significance assessed at *p* < 0.05.

## 5. Conclusions

In conclusion, the present study provides molecular evidence for the occurrence and genetic characterization of *M. cartusiana* in Pakistan based on mitochondrial markers (COI and 16S rRNA), together with haplotype network reconstruction, phylogenetic analyses, and neutrality tests. The results suggest a historical pattern of demographic expansion in *M. cartusiana*, potentially associated with post-glacial range expansion, followed by more recent dispersal events likely facilitated by human activities. Notably, the FST analyses indicate that the Pakistani population shows the greatest genetic affinity with Italian and Central European populations. This pattern is consistent with, though not conclusive proof of, a Southern or Central European origin, and may reflect human-mediated jump-dispersal via agricultural trade routes. Future analyses incorporating nuclear markers and explicit colonization scenario testing would be necessary to formally identify the introduction source. From an applied perspective, such genetic structuring may promote the development of local adaptations, with implications for pest management and may enable the identification of invasion sources through molecular tracing. Future research integrating the complete mitochondrial genome of *M. cartusiana* with additional nuclear genetic markers will be essential to further test hypotheses based on mitochondrial lineages and to achieve a more comprehensive understanding of gene flow, population connectivity, and the evolutionary dynamics of this species.

## Figures and Tables

**Table 1:** Diversity and neutrality indices for *M. cartusiana* (COI).

Indices	COI (555 bp)
No. of isolates	189
No. of mutations	58
Parsimony informative sites	35
No. of segregating (polymorphic) sites	53
No. of haplotypes	52
Haplotype diversity (Hd)	0.946
Nucleotide diversity (π)	0.010
Tajima’s D	−1.428
Fu’s Fs	−29.776
Fu and Li’s D	−3.281
Fu and Li’s F	−2.890
Average number of pairwise nucleotide differences (k)	5.256

**Table 2:** Diversity and neutrality indices for *M. cartusiana* (16S rRNA).

Indices	16S rRNA (269 bp)
No. of isolates	84
No. of mutations	18
Parsimony informative sites	14
No. of segregating (polymorphic) sites	18
No. of haplotypes	14
Haplotype diversity (Hd)	0.831
Nucleotide diversity (pi)	0.01253
Tajima’s D	−0.20586
Fu’s Fs	−1.263
Fu and Li’s D	−0.16487
Fu and Li’s F	−0.21434
Average number of pairwise nucleotide differences (k)	3.345

**Table 3:** Analysis of Molecular Variance (AMOVA) of *M. cartusiana* based on COI sequences.

Source of Variation	Df	Sum of Squares	Variance Components	Percentage of Variation	Significance
Among populations	14	161.748	0.8776	29.98%	*p* < 0.0001
Within populations	174	356.623	2.0495	70.02%	*p* < 0.0001
Total	188	518.370	2.9271	100%	*p* < 0.0001

**Table 4:** Analysis of Molecular Variance (AMOVA) based on the 16S rRNA gene of *M. cartusiana*.

Source of Variation	df	Sum of Squares	Variance Components	Percentage of Variation	Significance
Among populations	9	83.215	1.09478	51.40%	*p* < 0.001
Within populations	74	76.606	1.03522	48.60%	*p* < 0.001
Total	83	159.821	2.13001	100%	*p* < 0.001

**Table 5:** Geographic coordinates of sampling sites and distribution of *Monacha cartusiana*.

Site ID	Locality	Voucher (Catalog No.)	Latitude (N)	Longitude (E)	*M. cartusiana* (n)/ Total No.
S1	Haripur	HU-Zoo-NB-2024/01	34.086	72.95	129/1168
S2	Abbottabad	HU-Zoo-NB-2024/02	34.145	73.363	92/1142
S3	Battagram	HU-Zoo-NB-2024/03	34.557	73.17	85/1135
S4	Shinkiari	HU-Zoo-NB-2024/04	34.354	72.997	74/1155

**Table 6:** Primers, sequences and PCR conditions for amplification of *COI* and *16S rRNA* genes.

Gene	Primer Sequences (5′–3′)	PCR Conditions (35 cycles)	Amplicon Size	References
*COI*	F: GGTCAACAAATCATAAAGATATTGG R: TAAACTTCAGGGTGACCAAAAAATCA	94 °C 5 min; 94 °C 30 s; 48 °C 30 s; 72 °C 1 min; final 72 °C 5 min	~700 bp	[60]
*16S rRNA*	F: CGCCTGTTTATCAAAAACAT R: CCGGTCTGAACTCAGATCACGT	96 °C 2 min; 94 °C 30 s; 45 °C 60 s; 72 °C 2 min; final 72 °C 5 min	~400 bp	[61]

## Data Availability

All newly generated sequences produced in this study have been deposited in the NCBI GenBank public repository. COI sequences are available under accession numbers PX591882–PX591885, and 16S rRNA sequences under accession numbers PX591889–PX591892. All additional data used in this manuscript are available in the Supplementary File.

## Supplementary Materials

The following supporting information can be downloaded at: https://www.mdpi.com/article/10.3390/ijms27104318/s1, Table S1 to S4.

## Abbreviations

The following abbreviations are used in this manuscript:

PCR	Polymerase Chain Reaction
AMOVA	Analysis of Molecular Variance
TCS	Templeton Ceandall and Sing
FST	Fixation Index
