# Supplementary material for: Phylogeography and Population Structure of the Invasive Land Snail Monacha cartusiana
Source: Int J Mol Sci. 2026 May 12;27(10):4318. doi: 10.3390/ijms27104318 (PMC13206854; doi:10.3390/ijms27104318)
Supplement: Supplementary file 1 [file ijms-27-04318-s001.zip › ijms-4231464-supplementary.pdf]

Table S1: Various haplotypes of *Monacha cartusiana* identified in the current study based on the *COI* gene

Haplotype	No. of sequences	Accession numbers
**Hap_1**	5	PX591882, PX591884, PX591885 (Pakistan), PP947922, PP947920 (Italy)
**Hap_2**	26	PX591883 (Pakistan), PP947921, PP947919 (Italy), PP216511 (UK), PQ383793, PQ383792, PQ383772, PQ383767, PQ383742, PQ383695, PQ383666, PQ383659, PQ383640, PQ383634, MH203968, MH203967 (Germany), MT947682 (Austria), MT947665 (Czech Republic), MT947661, MT947652, MT947646, KF986836 (France), MH203966, MH203965 (Bosnia), KX258418 (Poland), KX507235 (Spain)
**Hap_3**	1	PP947923 (Italy)
**Hap_4**	12	PP947914 to PP947916, PP947911, PP947907 to PP947909, KX258407 to KX258409, KM247381, KM247380 (Poland)
**Hap_5**	20	PP947913, PP947912, PP947910, MT947683, KX258403 to KX258406, KM247382, KM247379 (Poland), MT947671, MT947662, MT947657 to MT947659, MT947654, MT947649 (Czech Republic), MH203987 to MH203989 (Slovakia),
**Hap_6**	3	PP947906.1, ON332654, ON332652 (France)
**Hap_7**	4	PP216510 (Hungary), MT947680, MT947678, MT947643 (Czech Republic)
**Hap_8**	1	PP216507 (Germany)
**Hap_9**	3	PP216506, PQ383705, PQ383699 (Germany)
**Hap_10**	18	PQ383783 to PQ383791, PQ383778.1, PQ383771, PQ383713, PQ383712, PQ383709, PQ383667, PQ383649, PQ383647, PQ383646, PQ383624 (Germany)
**Hap_11**	10	PQ383781, PQ383761, PQ383752, PQ383751, PQ383722, PQ383665, PQ383653, PQ383645, PQ383637, PQ383635 (Germany)

**Hap_12**	2	PQ383757, PQ383671 (Germany)
**Hap_13**	1	PQ383636 (Germany)
**Hap_14**	1	ON332655 (France)
**Hap_15**	1	ON332653 (France)
**Hap_16**	1	MT947691 (Slovakia)
**Hap_17**	1	MT947685 (Czech Republic)
**Hap_18**	1	MT947681 (France)
**Hap_19**	2	MT947674 (Czech Republic), KM247378 (Poland)
**Hap_20**	1	MT947672 (Czech Republic)
**Hap_21**	1	MT947670 (Slovenia)
**Hap_22**	1	MT947669 (Slovakia)
**Hap_23**	1	MT947666 (Slovenia)
**Hap_24**	3	MT947664 (Czech Republic), MH203983, MH203982 (Austria)
**Hap_25**	1	MT947663 (Czech Republic)
**Hap_26**	1	MT947648 (Croatia)
**Hap_27**	1	MT947647 (France)
**Hap_28**	1	MT947645 (Germany)
**Hap_29**	1	MG585400 (Spain)
**Hap_30**	1	MH203998 (Germany)

**Hap_31**	5	MH203993 to MH203997 (Germany)
**Hap_32**	1	MH203992 (Germany)
**Hap_33**	2	MH203991, MH203990 (Germany)
**Hap_34**	1	MH203986 (Austria)
**Hap_35**	2	MH203985, MH203984 (Austria)
**Hap_36**	1	MH203981 (Hungary)
**Hap_37**	1	MH203980 (Hungary)
**Hap_38**	4	MH203976 to MH203979 (Hungary)
**Hap_39**	7	MH203975 (Croatia), MH203964, MH203963 (Bosnia), KX258411 to KX258414 (Poland)
**Hap_40**	1	MH203974 (Kosovo)
**Hap_41**	3	MH203973 to MH203971 (Kosovo)
**Hap_42**	2	MH203970, MH203969 (Bosnia)
**Hap_43**	4	MH203959 to MH203962 (Bosnia)
**Hap_44**	3	MH203958 (Bosnia), KX258416, KX258415 (Poland)
**Hap_45**	1	MH203957 (Bosnia)
**Hap_46**	14	MH203948 to MH203956, KX258393 to KX258397 (Poland)
**Hap_47**	1	KX258417 (Poland)
**Hap_48**	1	KX258410 (Poland)
**Hap_49**	5	KX258398 to KX258402 (Poland)

**Hap_50**	2	KX507189 (Italy), KM247389 (Poland)
**Hap_51**	1	KM247377 (Poland)
**Hap_52**	1	KM247376 (Poland)

Table S2: Various haplotypes of *Monacha cartusiana* identified in the current study based on the *16S rRNA* gene

**Haplotype**	**Number of sequences**	**Accession numbers (countries)**
Hap_1	8	PX591889, PX591890 (Pakistan), MH204096 to MH204098 (Slovakia), MH204091 to MH204093 (Austria)
Hap_2	4	PX591891 (Pakistan), MH204087 to MH204089 (Kosovo)
Hap_3	3	PX591892 (Pakistan), MH204085, MH204084 (Bosnia)
Hap_ 4	25	MG585433 (Spain), MH204070 to MH204081 (Germany), MH204063 to MH204069 (Bosnia), KX258306, KX258305, KM247397 (Poland), KX495378, AY741416 (Italy)
Hap_5	2	MH204095, MH204094 (Austria)
Hap_6	1	MH204090 (Kosovo)
Hap_7	1	MH204086 (Kosovo)
Hap_8	4	MH204083, MH204082 (Bosnia), KX258303, KX258302 (Poland)
Hap_9	20	MH204047 to MH204062, KX258288 to KX258291 (Poland)
Hap_10	1	MH204046 (Poland)
Hap_11	1	KX258307 (Poland)
Hap_12	1	KX258304 (Poland)

Hap_13	11	KX258292 to KX258301, KM247391 (Poland)
Hap_14	2	KX495429, KJ458540 (Spain)

Table S3: Detailed information of COI gene sequences in *Monacha cartusiana* isolates from various countries reported in NCBI (https://www.ncbi.nlm.nih.gov)

S. n.	Accession No.	Source	Isolate / Voucher / Strain	Country	Sequence length	Year	Position	Authorities
1	PX591882	mitochondrion	sample_2	Pakistan	626bp	2025	1-626	sequence from the current study
2	PX591883	mitochondrion	sample_3	Pakistan	655bp	2025	1-655	sequence from the current study
3	PX591884	mitochondrion	sample_4	Pakistan	630bp	2025	1-630	sequence from the current study
4	PX591885	mitochondrion	sample_5	Pakistan	624bp	2025	1-624	sequence from the current study
5	PP947923	mitochondrion	Que5	Italy	672 bp	2025	1–672	Manganelli et al., Unpublished; Direct Submission 2024

6	PP947922	mitochondrion	Que4	Italy	672 bp	2025	1–672	Manganelli et al., Unpublished; Direct Submission 2024
7	PP947921	mitochondrion	Que3	Italy	672 bp	2025	1–672	Manganelli et al., Unpublished; Direct Submission 2024
8	PP947920	mitochondrion	Que2	Italy	672 bp	2025	1–672	Manganelli et al., Unpublished; Direct Submission 2024
9	PP947919	mitochondrion	Que1	Italy	672 bp	2025	1–672	Manganelli et al., Unpublished; Direct Submission 2024
10	PP947916	mitochondrion	Wro24	Poland	672 bp	2025	1–672	Manganelli et al., Unpublished; Direct Submission 2024
11	PP947915	mitochondrion	Wro23	Poland	672 bp	2025	1–672	Manganelli et al., Unpublished; Direct Submission 2024
12	PP947914	mitochondrion	Wro22	Poland	672 bp	2025	1–672	Manganelli et al., Unpublished; Direct Submission 2024
13	PP947913	mitochondrion	Wro20	Poland	672 bp	2025	1–672	Manganelli et al., Unpublished; Direct Submission 2024
14	PP947912	mitochondrion	Wro15	Poland	672 bp	2025	1–672	Manganelli et al., Unpublished; Direct Submission 2024
15	PP947911	mitochondrion	Wro14	Poland	672 bp	2025	1..672	Manganelli et al., Unpublished; Direct Submission 25-JUN-2024
16	PP947910	mitochondrion	Wro13	Poland	672 bp	2025	1..672	Manganelli et al., Unpublished; Direct Submission 25-JUN-2024
17	PP947909	mitochondrion	Wro12	Poland	672 bp	2025	1..672	Manganelli et al., Unpublished; Direct Submission 25-JUN-2024
18	PP947908	mitochondrion	Wro11	Poland	672 bp	2025	1..672	Manganelli et al., Unpublished; Direct Submission 25-JUN-2024
19	PP947907	mitochondrion	Wro10	Poland	672 bp	2025	1..672	Manganelli et al., Unpublished; Direct Submission 25-JUN-2024
20	PP947906	mitochondrion	Cur5	France	672 bp	2025	1..672	Manganelli et al., Unpublished; Direct Submission (25-JUN-2024)
21	PP216511	mitochondrion	D1032 / SMNG:p25238	United Kingdom	655 bp	2024	1..655	Williams et al. 2024; Direct Submission (26-JAN-2024)

22	PP216510	mitochondrion	D1031 / SMNG:p3066B	Hungary	655 bp	2024	1..655	Williams et al. 2024; Direct Submission (26-JAN-2024)
23	PP216507	mitochondrion	D1027 / SMNG:p25229-B-4-6	Germany	655 bp	2024	1..655	Williams et al. 2024; Direct Submission (26-JAN-2024)
24	PP216506	mitochondrion	D1028 / SMNG:p25229-B-4-8	Germany	655 bp	2024	1..655	Williams et al. 2024; Direct Submission (26-JAN-2024)
25	PQ383793	mitochondrion	MVTIS33351	Germany	595 bp	2024	1..595	Scheler, A. (Unpublished); Direct Submission (26-SEP-2024)
26	PQ383792	mitochondrion	MVTIS33346	Germany	595 bp	2024	1..595	Scheler, A. (Unpublished); Direct Submission (26-SEP-2024)
27	PQ383791	mitochondrion	MVTIS33338	Germany	595 bp	2024	1..595	Scheler, A. (Unpublished); Direct Submission (26-SEP-2024)
28	PQ383790	mitochondrion	MVTIS33349	Germany	595 bp	2024	1..595	Scheler, A. (Unpublished); Direct Submission (26-SEP-2024)
29	PQ383789	mitochondrion	MVTIS33355	Germany	595 bp	2024	1..595	Scheler, A. (Unpublished); Direct Submission (26-SEP-2024)
30	PQ383788	mitochondrion	MVTIS33357	Germany	595 bp	2024	1..595	Scheler, A. (Unpublished); Direct Submission (26-SEP-2024)
31	PQ383787	mitochondrion	MVTIS33360	Germany	595 bp	2024	1..595	Scheler, A. (Unpublished); Direct Submission (26-SEP-2024)
32	PQ383786	mitochondrion	MVTIS33361	Germany	595 bp	2024	1..595	Scheler, A. (Unpublished); Direct Submission (26-SEP-2024)
33	PQ383785	mitochondrion	MVTIS33364	Germany	595 bp	2024	1..595	Scheler, A. (Unpublished); Direct Submission (26-SEP-2024)
34	PQ383783	mitochondrion	MVTIS33374	Germany	595 bp	2024	1..595	Scheler, A. (Unpublished); Direct Submission (26-SEP-2024)
35	PQ383781	mitochondrion	AS128	Germany	585 bp	2024	1..585	Scheler, A. (Unpublished); Direct Submission (26-SEP-2024)

36	PQ383778	mitochondrion	AS125	Germany	585 bp	2024	1..585	Scheler, A. (Unpublished); Direct Submission (26-SEP-2024)
37	PQ383772	mitochondrion	AS99	Germany	585 bp	2024	1..585	Scheler, A. (Unpublished); Direct Submission (26-SEP-2024)
38	PQ383771	mitochondrion	AS98	Germany	585 bp	2024	1..585	Scheler, A. (Unpublished); Direct Submission (26-SEP-2024)
39	PQ383767	mitochondrion	AS93	Germany	585 bp	2024	1..585	Scheler, A. (Unpublished); Direct Submission (26-SEP-2024)
40	PQ383761	mitochondrion	AS112	Germany	585 bp	2024	1..585	Scheler, A. (Unpublished); Direct Submission (26-SEP-2024)
41	PQ383757	mitochondrion	AS108	Germany	585 bp	2024	1..585	Scheler, A. (Unpublished); Direct Submission (26-SEP-2024)
42	PQ383752	mitochondrion	AS187	Germany	585 bp	2024	1..585	Scheler, A. (Unpublished); Direct Submission (26-SEP-2024)
43	PQ383751	mitochondrion	AS185	Germany	585 bp	2024	1..585	Scheler, A. (Unpublished); Direct Submission (26-SEP-2024)
44	PQ383742	mitochondrion	AS141	Germany	585 bp	2024	1..585	Scheler, A. (Unpublished); Direct Submission (26-SEP-2024)
45	PQ383722	mitochondrion	AS63	Germany	585 bp	2024	1..585	Scheler, A. (Unpublished); Direct Submission (26-SEP-2024)
46	PQ383713	mitochondrion	AS159	Germany	585 bp	2024	1..585	Scheler, A. (Unpublished); Direct Submission (26-SEP-2024)
47	PQ383712	mitochondrion	AS157	Germany	585 bp	2024	1..585	Scheler, A. (Unpublished); Direct Submission (26-SEP-2024)
48	PQ383709	mitochondrion	AS92	Germany	585 bp	2024	1..585	Scheler, A. (Unpublished); Direct Submission (26-SEP-2024)
49	PQ383705	mitochondrion	AS19	Germany	585 bp	2024	1..585	Scheler, A. (Unpublished); Direct Submission (26-SEP-2024)
50	PQ383699	mitochondrion	AS153	Germany	585 bp	2024	1..585	Scheler, A. (Unpublished); Direct Submission (26-SEP-2024)
51	PQ383695	mitochondrion	AS181	Germany	585 bp	2024	1..585	Scheler, A. (Unpublished); Direct Submission (26-SEP-2024)

52	PQ383671	mitochondrion	AS52	Germany	585 bp	2024	1..585	Scheler, A. (Unpublished); Direct Submission (26-SEP-2024)
53	PQ383667	mitochondrion	AS151	Germany	585 bp	2024	1..585	Scheler, A. (Unpublished); Direct Submission (26-SEP-2024)
54	PQ383666	mitochondrion	AS150	Germany	585 bp	2024	1..585	Scheler, A. (Unpublished); Direct Submission (26-SEP-2024)
55	PQ383665	mitochondrion	AS86	Germany	585 bp	2024	1..585	Scheler, A. (Unpublished); Direct Submission (26-SEP-2024)
56	PQ383659	mitochondrion	AS41	Germany	585 bp	2024	1..585	Scheler, A. (Unpublished); Direct Submission (26-SEP-2024)
57	PQ383653	mitochondrion	AS31	Germany	585 bp	2024	1..585	Scheler, A. (Unpublished); Direct Submission (26-SEP-2024)
58	PQ383649	mitochondrion	AS27	Germany	585 bp	2024	1..585	Scheler, A. (Unpublished); Direct Submission (26-SEP-2024)
59	PQ383647	mitochondrion	AS25	Germany	585 bp	2024	1..585	Scheler, A. (Unpublished); Direct Submission (26-SEP-2024)
60	PQ383646	mitochondrion	AS24	Germany	585 bp	2024	1..585	Scheler, A. (Unpublished); Direct Submission (26-SEP-2024)
61	PQ383645	mitochondrion	AS23	Germany	585 bp	2024	1..585	Scheler, A. (Unpublished); Direct Submission (26-SEP-2024)
62	PQ383640	mitochondrion	AS69	Germany	585 bp	2024	1..585	Scheler, A. (Unpublished); Direct Submission (26-SEP-2024)
63	PQ383637	mitochondrion	AS76	Germany	585 bp	2024	1..585	Scheler, A. (Unpublished); Direct Submission (26-SEP-2024)
64	PQ383636	mitochondrion	AS75	Germany	585 bp	2024	1..585	Scheler, A. (Unpublished); Direct Submission (26-SEP-2024)
65	PQ383635	mitochondrion	AS123	Germany	585 bp	2024	1..585	Scheler, A. (Unpublished); Direct Submission (26-SEP-2024)
66	PQ383634	mitochondrion	AS122	Germany	585 bp	2024	1..585	Scheler, A. (Unpublished); Direct Submission (26-SEP-2024)
67	PQ383624	mitochondrion	AS1	Germany	585 bp	2024	1..585	Scheler, A. (Unpublished); Direct Submission (26-SEP-2024)

68	ON33265 5	mitochondrio n	Cur 4	France	684 bp	2022	1..684	Pienkowska, J.R. et al., Eur Zool J 89(1), 966-990 (2022); Direct Submission (25-APR-2022)
69	ON33265 4	mitochondrio n	Cur 3	France	684 bp	2022	1..684	Pienkowska, J.R. et al., Eur Zool J 89(1), 966-990 (2022); Direct Submission (25-APR-2022)
70	ON33265 3	mitochondrio n	Cur 2	France	684 bp	2022	1..684	Pienkowska, J.R. et al., Eur Zool J 89(1), 966-990 (2022); Direct Submission (25-APR-2022)
71	ON33265 2	mitochondrio n	Cur 1	France	684 bp	2022	1..684	Pienkowska, J.R. et al., Eur Zool J 89(1), 966-990 (2022); Direct Submission (25-APR-2022)
72	MT94769 1	mitochondrio n	VR1	Slovakia	627 bp	2022	1..627	Cejka, T. et al., Malacol Bohemoslov 19, 71-106 (2020); Direct Submission (31-AUG-2020)
73	MT94768 5	mitochondrio n	T1	Czech Republic	627 bp	2022	1..627	Cejka, T. et al., Malacol Bohemoslov 19, 71-106 (2020); Direct Submission (31-AUG-2020)
74	MT94768 3	mitochondrio n	STT1	Poland	627 bp	2022	1..627	Cejka, T. et al., Malacol Bohemoslov 19, 71-106 (2020); Direct Submission (31-AUG-2020)
75	MT94768 2	mitochondrio n	RK2	Austria	627 bp	2022	1..627	Cejka, T. et al., Malacol Bohemoslov 19, 71-106 (2020); Direct Submission (31-AUG-2020)
76	MT94768 1	mitochondrio n	RGN1	France	627 bp	2022	1..627	Cejka, T. et al., Malacol Bohemoslov 19, 71-106 (2020); Direct Submission (31-AUG-2020)
77	MT94768 0	mitochondrio n	R1	Czech Republic	576 bp	2022	1..576	Cejka, T. et al., Malacol Bohemoslov 19, 71-106 (2020); Direct Submission (31-AUG-2020)

78	MT947678	mitochondrion	PCM1	Czech Republic	627 bp	2022	1..627	Cejka, T. et al., Malacol Bohemoslov 19, 71-106 (2020); Direct Submission (31-AUG-2020)
79	MT947674	mitochondrion	OL1	Czech Republic	627 bp	2022	1..627	Cejka, T. et al., Malacol Bohemoslov 19, 71-106 (2020); Direct Submission (31-AUG-2020)
80	MT947672	mitochondrion	N1	Czech Republic	627 bp	2022	1..627	Cejka, T. et al., Malacol Bohemoslov 19, 71-106 (2020); Direct Submission (31-AUG-2020)
81	MT947671	mitochondrion	MS1	Czech Republic	627 bp	2022	1..627	Cejka, T. et al., Malacol Bohemoslov 19, 71-106 (2020); Direct Submission (31-AUG-2020)
82	MT947670	mitochondrion	MRI1	Slovenia	627 bp	2022	1..627	Cejka, T. et al., Malacol Bohemoslov 19, 71-106 (2020); Direct Submission (31-AUG-2020)
83	MT947669	mitochondrion	Mar-01	Slovakia	627 bp	2022	1..627	Cejka, T. et al., Malacol Bohemoslov 19, 71-106 (2020); Direct Submission (31-AUG-2020)
84	MT947666	mitochondrion	LJU1	Slovenia	627 bp	2022	1..627	Cejka, T. et al., Malacol Bohemoslov 19, 71-106 (2020); Direct Submission (31-AUG-2020)
85	MT947665	mitochondrion	KOL1	Czech Republic	627 bp	2022	1..627	Cejka, T. et al., Malacol Bohemoslov 19, 71-106 (2020); Direct Submission (31-AUG-2020)
86	MT947664	mitochondrion	KK1	Czech Republic	627 bp	2022	1..627	Cejka, T. et al., Malacol Bohemoslov 19, 71-106 (2020); Direct Submission (31-AUG-2020)
87	MT947663	mitochondrion	KH1	Czech Republic	627 bp	2022	1..627	Cejka, T. et al., Malacol Bohemoslov 19, 71-106 (2020); Direct Submission (31-AUG-2020)

88	MT947662	mitochondrion	KEB1	Czech Republic	627 bp	2022	1..627	Cejka, T. et al., Malacol Bohemoslov 19, 71-106 (2020); Direct Submission (31-AUG-2020)
89	MT947661	mitochondrion	JQE1	Czech Republic	627 bp	2022	1..627	Cejka, T. et al., Malacol Bohemoslov 19, 71-106 (2020); Direct Submission (31-AUG-2020)
90	MT947659	mitochondrion	IV1	Czech Republic	627 bp	2022	1..627	Cejka, T. et al., Malacol Bohemoslov 19, 71-106 (2020); Direct Submission (31-AUG-2020)
91	MT947658	mitochondrion	IE1	Czech Republic	627 bp	2022	1..627	Cejka, T. et al., Malacol Bohemoslov 19, 71-106 (2020); Direct Submission (31-AUG-2020)
92	MT947657	mitochondrion	HA1	Germany	627 bp	2022	1..627	Cejka, T. et al., Malacol Bohemoslov 19, 71-106 (2020); Direct Submission (31-AUG-2020)
93	MT947654	mitochondrion	DA1	Czech Republic	627 bp	2022	1..627	
94	MT947652	mitochondrion	CNN1	France	627 bp	2022	1..627	Cejka, T. et al., Malacol Bohemoslov 19, 71-106 (2020); Direct Submission (31-AUG-2020)
95	MT947649	mitochondrion	BY2	Czech Republic	627 bp	2022	1..627	Cejka, T. et al., Malacol Bohemoslov 19, 71-106 (2020); Direct Submission (31-AUG-2020)
96	MT947648	mitochondrion	BR1	Croatia	627 bp	2022	1..627	Cejka, T. et al., Malacol Bohemoslov 19, 71-106 (2020); Direct Submission (31-AUG-2020)
97	MT947647	mitochondrion	BNX1	France	627 bp	2022	1..627	Cejka, T. et al., Malacol Bohemoslov 19, 71-106 (2020); Direct Submission (31-AUG-2020)
98	MT947646	mitochondrion	BER1	France	627 bp	2022	1..627	Cejka, T. et al., Malacol Bohemoslov 19, 71-106 (2020); Direct Submission (31-AUG-2020)

99	MT947645	mitochondrion	BBN1	Germany	625 bp	2022	1..625	Cejka, T. et al., Malacol Bohemoslov 19, 71-106 (2020); Direct Submission (31-AUG-2020)
100	MT947643	mitochondrion	B2	Czech Republic	627 bp	2022	1..627	Cejka, T. et al., Malacol Bohemoslov 19, 71-106 (2020); Direct Submission (31-AUG-2020)
101	MG585400	mitochondrion	EHUMC-2080	Spain	660 bp	2019	1..660	Caro, A. et al., Mol. Phylogenet. Evol. 139, 106570 (2019); Direct Submission (22-NOV-2017)
102	MH203998	mitochondrion	Han_1	Germany	627 bp	2018	1..627	Pienkowska, J.R. et al., Folia Malacol 26 (2), 103-120 (2018); Direct Submission (12-APR-2018)
103	MH203997	mitochondrion	Bon_5	Germany	627 bp	2018	1..627	Pienkowska, J.R. et al., Folia Malacol 26 (2), 103-120 (2018); Direct Submission (12-APR-2018)
104	MH203996	mitochondrion	Bon_4	Germany	627 bp	2018	1..627	Pienkowska, J.R. et al., Folia Malacol 26 (2), 103-120 (2018); Direct Submission (12-APR-2018)
105	MH203995	mitochondrion	Bon_3	Germany	627 bp	2018	1..627	Pienkowska, J.R. et al., Folia Malacol 26 (2), 103-120 (2018); Direct Submission (12-APR-2018)
106	MH203994	mitochondrion	Bon_2	Germany	627 bp	2018	1..627	Pienkowska, J.R. et al., Folia Malacol 26 (2), 103-120 (2018); Direct Submission (12-APR-2018)
107	MH203993	mitochondrion	Bon_1	Germany	627 bp	2018	1..627	Pienkowska, J.R. et al., Folia Malacol 26 (2), 103-120 (2018); Direct Submission (12-APR-2018)
108	MH203992	mitochondrion	Col_2	Germany	627 bp	2018	1..627	Pienkowska, J.R. et al., Folia Malacol 26 (2), 103-120 (2018); Direct Submission (12-APR-2018)
109	MH203991	mitochondrion	Col_4	Germany	627 bp	2018	1..627	

110	MH203990	mitochondrion	Col_1	Germany	627 bp	2018	1..627	Pienkowska, J.R. et al., Folia Malacol 26 (2), 103-120 (2018); Direct Submission (12-APR-2018)
111	MH203989	mitochondrion	Cat_3	Slovakia	627 bp	2018	1..627	Pienkowska, J.R. et al., Folia Malacol 26 (2), 103-120 (2018); Direct Submission (12-APR-2018)
112	MH203988	mitochondrion	Cat_2	Slovakia	627 bp	2018	1..627	Pienkowska, J.R. et al., Folia Malacol 26 (2), 103-120 (2018); Direct Submission (12-APR-2018)
113	MH203987	mitochondrion	Cat_1	Slovakia	627 bp	2018	1..627	Pienkowska, J.R. et al., Folia Malacol 26 (2), 103-120 (2018); Direct Submission (12-APR-2018)
114	MH203986	mitochondrion	Mar_3	Austria	627 bp	2018	1..627	Pienkowska, J.R. et al., Folia Malacol_
115	MH203985	mitochondrion	Mar_5	Austria	627 bp	2018	1..627	Pienkowska, J.R. et al., Folia Malacol 26 (2), 103-120 (2018); Direct Submission (12-APR-2018)
116	MH203984	mitochondrion	Mar_2	Austria	627 bp	2018	1..627	Pienkowska, J.R. et al., Folia Malacol 26 (2), 103-120 (2018); Direct Submission (12-APR-2018)
117	MH203983	mitochondrion	Mar_4	Austria	627 bp	2018	1..627	Pienkowska, J.R. et al., Folia Malacol 26 (2), 103-120 (2018); Direct Submission (12-APR-2018)
118	MH203982	mitochondrion	Mar_1	Austria	627 bp	2018	1..627	Pienkowska, J.R. et al., Folia Malacol 26 (2), 103-120 (2018); Direct Submission (1_
119	MH203981	mitochondrion	B_2	Hungary	627 bp	2018	1..627	Pienkowska, J.R. et al., Folia Malacol 26 (2), 103-120 (2018); Direct Submission (12-APR-2018)
120	MH203980	mitochondrion	B_1	Hungary	627 bp	2018	1..627	Pienkowska, J.R. et al., Folia Malacol 26 (2), 103-120 (2018); Direct Submission (12-APR-2018)

121	MH20397 9	mitochondrio n	B_4	Hungary	627 bp	2018	1..627	Pienkowska, J.R. et al., Folia Malacol 26 (2), 103-120 (2018); Direct Submission (12-APR-2018)
122	MH20397 8	mitochondrio n	B_3	Hungary	627 bp	2018	1..627	Pienkowska, J.R. et al., Folia Malacol 26 (2), 103-120 (2018); Direct Submission (12-APR-2018)
123	MH20397 7	mitochondrio n	B_7	Hungary	627 bp	2018	1..627	Pienkowska, J.R. et al., Folia Malacol 26 (2), 103-120 (2018); Direct Submission (12-APR-2018)
124	MH20397 6	mitochondrio n	B_6	Hungary	627 bp	2018	1..627	Pienkowska, J.R. et al., Folia Malacol 26 (2), 103-120 (2018); Direct Submission (12-APR-2018)
125	MH20397 5	mitochondrio n	Don_1	Croatia	627 bp	2018	1..627	Pienkowska, J.R. et al., Folia Malacol 26 (2), 103-120 (2018); Direct Submission (12-APR-2018)
126	MH20397 4	mitochondrio n	Kos_2	Kosovo	627 bp	2018	1..627	Pienkowska, J.R. et al., Folia Malacol 26 (2), 103-120 (2018); Direct Submission (12-APR-2018)
127	MH20397 3	mitochondrio n	Kos_1	Kosovo	627 bp	2018	1..627	Pienkowska, J.R. et al., Folia Malacol 26 (2), 103-120 (2018); Direct Submission (12-APR-2018)
128	MH20397 2	mitochondrio n	Kos_4	Kosovo	627 bp	2018	1..627	Pienkowska, J.R. et al., Folia Malacol 26 (2), 103-120 (2018); Direct Submission (12-APR-2018)
129	MH20397 1	mitochondrio n	Kos_3	Kosovo	627 bp	2018	1..627	Pienkowska, J.R. et al., Folia Malacol 26 (2), 103-120 (2018); Direct Submission (12-APR-2018)
130	MH20397 0	mitochondrio n	Bab_2	Bosnia	627 bp	2018	1..627	Pienkowska, J.R. et al., Folia Malacol 26 (2), 103-120 (2018); Direct Submission (12-APR-2018)

131	MH20396 9	mitochondrio n	Bab_1	Bosnia	627 bp	2018	1..627	Pienkowska, J.R. et al., Folia Malacol 26 (2), 103-120 (2018); Direct Submission (12-APR-2018)
132	MH20396 8	mitochondrio n	Col_6	Germany	627 bp	2018	1..627	Pienkowska, J.R. et al., Folia Malacol 26 (2), 103-120 (2018); Direct Submission (12-APR-2018)
133	MH20396 7	mitochondrio n	Col_5	Germany	627 bp	2018	1..627	Pienkowska, J.R. et al., Folia Malacol 26 (2), 103-120 (2018); Direct Submission (12-APR-2018)
134	MH20396 6	mitochondrio n	Jaj_1	Bosnia	627 bp	2018	1..627	Pienkowska, J.R. et al., Folia Malacol 26 (2), 103-120 (2018); Direct Submission (12-APR-2018)
135	MH20396 5	mitochondrio n	Pot_1	Bosnia	627 bp	2018	1..627	Pienkowska et al., 2018; Direct submission, 2018
136	MH20396 4	mitochondrio n	Pot_4	Bosnia	627 bp	2018	1..627	Pienkowska et al., 2018; Direct submission, 2018
137	MH20396 3	mitochondrio n	Pot_3	Bosnia	627 bp	2018	1..627	Pienkowska et al., 2018; Direct submission, 2018
138	MH20396 2	mitochondrio n	Bog_3	Bosnia	627 bp	2018	1..627	Pienkowska et al., 2018; Direct submission, 2018
139	MH20396 1	mitochondrio n	Bog_2	Bosnia	627 bp	2018	1..627	Pienkowska et al., 2018; Direct submission, 2018
140	MH20396 0	mitochondrio n	Bog_1	Bosnia	627 bp	2018	1..627	Pienkowska et al., 2018; Direct submission, 2018
141	MH20395 9	mitochondrio n	Pot_2	Bosnia	627 bp	2018	1..627	Pienkowska et al., 2018; Direct submission, 2018
142	MH20395 8	mitochondrio n	Bro_1	Bosnia	627 bp	2018	1..627	Pienkowska et al., 2018; Direct submission, 2018
143	MH20395 7	mitochondrio n	Kao_1	Bosnia	627 bp	2018	1..627	Pienkowska et al., 2018; Direct submission, 2018
144	MH20395 6	mitochondrio n	Lid_7	Poland	627 bp	2018	1..627	Pienkowska et al., 2018; Direct submission, 2018

145	MH20395 5	mitochondrio n	Lid_6	Poland	627 bp	2018	1..627	Pienkowska et al. 2018; Direct Submission 2018
146	MH20395 4	mitochondrio n	Lid_5	Poland	627 bp	2018	1..627	Pienkowska et al. 2018; Direct Submission 2018
147	MH20395 3	mitochondrio n	Lid_4	Poland	627 bp	2018	1..627	Pienkowska et al. 2018; Direct Submission 2018
148	MH20395 2	mitochondrio n	Lid_2	Poland	627 bp	2018	1..627	Pienkowska et al. 2018; Direct Submission 2018
149	MH20395 1	mitochondrio n	Lid_1	Poland	627 bp	2018	1..627	Pienkowska et al. 2018; Direct Submission 2018
150	MH20395 0	mitochondrio n	Wiet_11	Poland	627 bp	2018	1..627	Pienkowska et al. 2018; Direct Submission 2018
151	MH20394 9	mitochondrio n	Wiet_9	Poland	627 bp	2018	1..627	Pienkowska et al. 2018; Direct Submission 2018
152	MH20394 8	mitochondrio n	Wiet_7	Poland	627 bp	2018	1..627	Pienkowska et al. 2018; Direct Submission 2018
153	KX25841 8	mitochondrio n	Bel5	Poland	585 bp	2016	1..585	Pienkowska et al. 2016; Direct Submission 2016
154	KX25841 7	mitochondrio n	Bel4	Poland	585 bp	2016	1..585	Pienkowska et al. 2016; Direct Submission 2016
155	KX25841 6	mitochondrio n	Bel3	Poland	585 bp	2016	1..585	Pienkowska et al. 2016; Direct Submission 2016
156	KX25841 5	mitochondrio n	Bel2	Poland	585 bp	2016	1..585	Pienkowska et al. 2016; Direct Submission 2016
157	KX25841 4	mitochondrio n	BH1-8	Poland	585 bp	2016	1..585	Pienkowska et al. 2016; Direct Submission 2016
158	KX25841 3	mitochondrio n	BH1-7	Poland	585 bp	2016	1..585	Pienkowska et al. 2016; Direct Submission 2016
159	KX25841 2	mitochondrio n	BH1-5	Poland	585 bp	2016	1..585	Pienkowska et al. 2016; Direct Submission 2016
160	KX25841 1	mitochondrio n	BH1-4	Poland	585 bp	2016	1..585	Pienkowska et al. 2016; Direct Submission 2016

161	KX25841<br>0	mitochondrio<br>n	BH1-6	Poland	585 bp	2016	1..585	Pienkowska et al. 2016; Direct Submission 2016
162	KX25840<br>9	mitochondrio<br>n	Wro8	Poland	585 bp	2016	1..585	Pienkowska et al. 2016; Direct Submission 2016
163	KX25840<br>8	mitochondrio<br>n	Wro6	Poland	585 bp	2016	1..585	Pienkowska et al. 2016; Direct Submission 2016
164	KX25840<br>7	mitochondrio<br>n	Wro5	Poland	585 bp	2016	1..585	Pienkowska et al. 2016; Direct Submission 2016
165	KX25840<br>6	mitochondrio<br>n	Bel6	Poland	585 bp	2016	1..585	Pienkowska et al. 2016; Direct Submission
166	KX25840<br>5	mitochondrio<br>n	Bel1	Poland	585 bp	2016	1..585	Pienkowska et al. 2016; Direct Submission
167	KX25840<br>4	mitochondrio<br>n	Wro9	Poland	585 bp	2016	1..585	Pienkowska et al. 2016; Direct Submission
168	KX25840<br>3	mitochondrio<br>n	Wro7	Poland	585 bp	2016	1..585	Pienkowska et al. 2016; Direct Submission
169	KX25840<br>2	mitochondrio<br>n	Os5	Poland	585 bp	2016	1..585	Pienkowska et al. 2016; Direct Submission
170	KX25840<br>1	mitochondrio<br>n	Os4	Poland	585 bp	2016	1..585	Pienkowska et al. 2016; Direct Submission
171	KX25840<br>0	mitochondrio<br>n	Os3	Poland	585 bp	2016	1..585	Pienkowska et al. 2016; Direct Submission
172	KX25839<br>9	mitochondrio<br>n	Os2	Poland	585 bp	2016	1..585	Pienkowska et al. 2016; Direct Submission
173	KX25839<br>8	mitochondrio<br>n	Os1	Poland	585 bp	2016	1..585	Pienkowska et al. 2016; Direct Submission
174	KX25839<br>7	mitochondrio<br>n	Wiet1	Poland	585 bp	2016	1..585	Pienkowska et al. 2016; Direct Submission
175	KX25839<br>6	mitochondrio<br>n	KG5	Poland	585 bp	2016	1..585	Pienkowska et al. 2016; Direct Submission
176	KX25839<br>5	mitochondrio<br>n	KG4	Poland	585 bp	2016	1..585	Pienkowska et al. 2016; Direct Submission

177	KX25839 4	mitochondrio n	KG3	Poland	585 bp	2016	1..585	Pienkowska et al. 2016; Direct Submission
178	KX25839 3	mitochondrio n	KG2	Poland	585 bp	2016	1..585	Pienkowska et al. 2016; Direct Submission
179	KX50723 5	mitochondrio n	SP166	Spain	655 bp	2015	1..655	Neiber & Hausdorf 2015; Direct Submission
180	KX50718 9	mitochondrio n	ZMH 51710-1594	Italy	655 bp	2015	1..655	Neiber & Hausdorf 2015; Direct Submission
181	KM24738 9	mitochondrio n	COI 15	Poland	635 bp	2015	1..635	Pienkowska et al. 2015; Direct Submission
182	KM24738 2	mitochondrio n	COI 8	Poland	635 bp	2015	1..635	Pienkowska et al. 2015; Direct Submission
183	KM24738 1	mitochondrio n	COI 7	Poland	635 bp	2015	1..635	Pienkowska et al. 2015; Direct Submission
184	KM24738 0	mitochondrio n	COI 6	Poland	635 bp	2015	1..635	Pienkowska et al. 2015; Direct Submission
185	KM24737 9	mitochondrio n	COI 5	Poland	635 bp	2015	1..635	Pienkowska et al. 2015; Direct Submission
186	KM24737 8	mitochondrio n	COI 4	Poland	635 bp	2015	1..635	Pienkowska et al. 2015; Direct Submission
187	KM24737 7	mitochondrio n	COI 3	Poland	635 bp	2015	1..635	Pienkowska et al. 2015; Direct Submission
188	KM24737 6	mitochondrio n	COI 2	Poland	635 bp	2015	1..635	Pienkowska et al. 2015; Direct Submission
189	KF986836	mitochondrio n	Mc	France	595 bp	2014	1..595	Dahirel et al., Unpublished; Direct Submission

Table S4: Detailed information of 16S rRNA gene sequences in *Monacha cartusiana* isolates from various countries reported in NCBI (https://www.ncbi.nlm.nih.gov)

S. no.	Accession number	Source	Isolate / Voucher / Strain	Country	Sequence length	Year	Position	Authorities
1	PX591889.1	mitochondrion		Pakistan	286	2025	1-286	Sequence of the current study
2	PX591890.1	mitochondrion		Pakistan	286	2025	1-287	Sequence of the current study
3	PX591891.1	mitochondrion		Pakistan	286	2025	1-288	Sequence of the current study
4	PX591892.1	mitochondrion		Pakistan	286	2025	1-289	Sequence of the current study
5	MG585433	mitochondrion	Voucher: EHUMC-2080	Spain	833 bp	2019	1–833	Caro et al. 2019; Direct Submission 2017
6	KX495429	mitochondrion	SP166	Spain	846 bp	2015	1–846	Pienkowska et al., 2018; Direct Submission 2018
7	KJ458540	mitochondrion	Mon_car166	Spain	409 bp	2014	1–409	Pienkowska et al. 2018; Direct Submission 2018
8	MH204098	mitochondrion	Cat_3	Slovakia	286 bp	2018	1–286	Pienkowska et al. 2018; Direct Submission 2018
9	MH204097	mitochondrion	Isolate: Cat_2	Slovakia	286 bp	2018	1–286	Pienkowska et al. 2018; Direct Submission 2018
10	MH204096	mitochondrion	Isolate: Cat_1	Slovakia	286 bp	2018	1–286	Pienkowska et al., 2018; Direct Submission 2018
11	MH204095	mitochondrion	Isolate: Mar_5	Austria	286 bp	2018	1–286	Pienkowska et al., 2018; Direct Submission 2018
12	MH204094	mitochondrion	Mar_2	Austria	286 bp	2018	1–286	Pienkowska et al., 2018; Direct Submission 2018

13	MH204093	mitochondrion	Mar_3	Austria	286 bp	2018	1–286	Pienkowska et al., 2018; Direct Submission 2018
14	MH204092	mitochondrion	Mar_4	Austria	286 bp	2018	1–286	Pienkowska et al., 2018; Direct Submission 2018
15	MH204091	mitochondrion	Mar_1	Austria	286 bp	2018	1–286	Pienkowska et al. 2018; Direct Submission 2018
16	MH204090	mitochondrion	Don_1	Kosovo	286 bp	2018	1–286	Pienkowska et al. 2018; Direct Submission 2018
17	MH204089	mitochondrion	Kos_2	Kosovo	286 bp	2018	1–286	Pienkowska et al. 2018; Direct Submission 2018
18	MH204088	mitochondrion	Kos_4	Kosovo	286 bp	2018	1–286	Pienkowska et al. 2018; Direct Submission 2018
19	MH204087	mitochondrion	Kos_1	Kosovo	286 bp	2018	1–286	Pienkowska et al. 2018; Direct Submission 2018
20	MH204086	mitochondrion	Kos_3	Kosovo	286 bp	2018	1–286	Pienkowska et al., 2018; Direct Submission
21	MH204085	mitochondrion	Jaj_1	Bosnia	286 bp	2018	1–286	Pienkowska et al., 2018; Direct Submission
22	MH204084	mitochondrion	Pot_1	Bosnia	286 bp	2018	1–286	Pienkowska et al., 2018; Direct Submission
23	MH204083	mitochondrion	Pot_4	Bosnia	286 bp	2018	1–286	Pienkowska et al., 2018; Direct Submission
24	MH204082	mitochondrion	Pot_3	Bosnia	286 bp	2018	1–286	Pienkowska et al., 2018; Direct Submission
25	MH204069	mitochondrion	Bab_2	Bosnia	286 bp	2018	1–286	Pienkowska et al., 2018; Direct Submission
26	MH204068	mitochondrion	Bab_1	Bosnia	286 bp	2018	1–286	Pienkowska et al., 2018; Direct Submission
27	MH204067	mitochondrion	Bog_3	Bosnia	286 bp	2018	1–286	Pienkowska et al., 2018; Direct Submission
28	MH204066	mitochondrion	Bog_2	Bosnia	286 bp	2018	1–286	Pienkowska et al., 2018; Direct Submission

29	MH204065	mitochondrion	Bog_1	Bosnia	286 bp	2018	1–286	Pienkowska et al., 2018; Direct Submission
30	MH204064	mitochondrion	Pot_2	Bosnia	286 bp	2018	1–286	Pienkowska et al., 2018; Direct Submission
31	MH204063	mitochondrion	Kao_1	Bosnia	286 bp	2018	1–286	Pienkowska et al., 2018; Direct Submission
32	MH204055	mitochondrion	Bro_1	Bosnia	286 bp	2018	1–286	Pienkowska et al., 2018; Direct Submission
33	MH204081	mitochondrion	Han_1	Germany	286 bp	2018	1–286	Pienkowska et al., 2018; Direct Submission
34	MH204080	mitochondrion	Bon_5	Germany	286 bp	2018	1–286	Pienkowska et al., 2018; Direct Submission
35	MH204079	mitochondrion	Bon_4	Germany	286 bp	2018	1–286	Pienkowska et al., 2018; Direct Submission 2018
36	MH204078	mitochondrion	Bon_3	Germany	286 bp	2018	1–286	Pienkowska et al., 2018; Direct Submission 2018
37	MH204077	mitochondrion	Bon_2	Germany	286 bp	2018	1–286	Pienkowska et al., 2018; Direct Submission 2018
38	MH204076	mitochondrion	Bon_1	Germany	286 bp	2018	1–286	Pienkowska et al., 2018; Direct Submission 2018
39	MH204075	mitochondrion	Col_3	Germany	287 bp	2018	1–287	Pienkowska et al., 2018; Direct Submission 2018
40	MH204074	mitochondrion	Col_6	Germany	287 bp	2018	1–287	Pienkowska et al., 2018; Direct Submission 2018
41	MH204073	mitochondrion	Col_5	Germany	286 bp	2018	1–286	Pienkowska et al., 2018; Direct Submission 2018
42	MH204072	mitochondrion	Col_2	Germany	286 bp	2018	1–286	Pienkowska et al., 2018; Direct Submission 2018
43	MH204071	mitochondrion	Col_4	Germany	286 bp	2018	1–286	Pienkowska et al., 2018; Direct Submission 2018
44	MH204070	mitochondrion	Col_1	Germany	286 bp	2018	1–286	Pienkowska et al., 2018; Direct Submission 2018

45	MH204062	mitochondrion	B_7	Hungary	287 bp	2018	1–287	Pienkowska et al., 2018; Direct Submission 2018
46	MH204061	mitochondrion	B_6	Hungary	287 bp	2018	1–287	Pienkowska et al., 2018; Direct Submission 2018
47	MH204060	mitochondrion	B_5	Hungary	287 bp	2018	1–287	Pienkowska et al., 2018; Direct Submission 2018
48	MH204059	mitochondrion	B_4	Hungary	287 bp	2018	1–287	Pienkowska et al., 2018; Direct Submission 2018
49	MH204058	mitochondrion	B_3	Hungary	287 bp	2018	1–287	Pienkowska et al., 2018; Direct Submission 2018
50	MH204057	mitochondrion	B_2	Hungary	287 bp	2018	1–287	Pienkowska et al., 2018; Direct Submission 2018
51	MH204056	mitochondrion	B_1	Hungary	287 bp	2018	1–287	Pienkowska et al., 2018; Direct Submission 2018
52	MH204054	mitochondrion	Lid_7	Poland	286 bp	2018	1–286	Pienkowska et al., 2018; Direct Submission 2018
53	MH204053	mitochondrion	Lid_6	Poland	286 bp	2018	1–286	Pienkowska et al., 2018; Direct Submission 2018
54	MH204052	mitochondrion	Lid_5	Poland	286 bp	2018	1–286	Pienkowska et al., 2018; Direct Submission 2018
55	MH204051	mitochondrion	Lid_4	Poland	286 bp	2018	1–286	Pienkowska et al., 2018; Direct Submission 2018
56	MH204050	mitochondrion	Lid_2	Poland	286 bp	2018	1–286	Pienkowska et al., 2018; Direct Submission 2018
57	MH204049	mitochondrion	Lid_1	Poland	286 bp	2018	1–286	Pienkowska et al., 2018; Direct Submission 2018
58	MH204048	mitochondrion	Wiet_11	Poland	286 bp	2018	1–286	Pienkowska et al., 2018; Direct Submission 2018
59	MH204047	mitochondrion	Wiet_9	Poland	286 bp	2018	1–286	Pienkowska et al., 2016; Direct Submission 2016
60	MH204046	mitochondrion	Wiet_7	Poland	284 bp	2018	1–284	Pienkowska et al., 2016; Direct Submission 2016

61	KX258307	mitochondrion	Bel6	Poland	269 bp	2016	1–269	Pienkowska et al., 2016; Direct Submission 2016
62	KX258306	mitochondrion	Bel5	Poland	269 bp	2016	1–269	Pienkowska et al., 2016; Direct Submission 2016
63	KX258305	mitochondrion	Bel4	Poland	269 bp	2016	1–269	Pienkowska et al., 2016; Direct Submission 2016
64	KX258304	mitochondrion	Bel1	Poland	269 bp	2016	1–269	Pienkowska et al., 2016; Direct Submission 2016
65	KX258303	mitochondrion	BH1-4	Poland	269 bp	2016	1–269	Pienkowska et al., 2016; Direct Submission 2016
66	KX258302	mitochondrion	BH1-6	Poland	269 bp	2016	1–269	Pienkowska et al., 2016; Direct Submission 2016
67	KX258301	mitochondrion	Wro9	Poland	269 bp	2016	1–269	Pienkowska et al., 2016; Direct Submission 2016
68	KX258300	mitochondrion	Wro8	Poland	269 bp	2016	1–269	Pienkowska et al., 2016; Direct Submission 2016
69	KX258299	mitochondrion	Wro7	Poland	Poland 269 bp	2016	1–269	Pienkowska et al., 2016; Direct Submission 2016
70	KX258298	mitochondrion	Wro6	Poland	269 bp	2016	1–269	Pienkowska et al., 2016; Direct Submission 2016
71	KX258297	mitochondrion	Wro5	Poland	269 bp	2016	1–269	Pienkowska et al., 2016; Direct Submission 2016
72	KX258296	mitochondrion	Os5	Poland	269 bp	2016	1–269	Pienkowska et al., 2016; Direct Submission 2016
73	KX258295	mitochondrion	Os4	Poland	269 bp	2016	1–269	Pienkowska et al., 2016; Direct Submission 2016
74	KX258294	mitochondrion	Os3	Poland	269 bp	2016	1–269	Pienkowska et al., 2016; Direct Submission 2016
75	KX258293	mitochondrion	Os2	Poland	269 bp	2016	1–269	Pienkowska et al., 2016; Direct Submission 2016
76	KX258292	mitochondrion	Os1	Poland	269 bp	2016	1–269	Pienkowska et al., 2016; Direct Submission 2016

77	KX258291	mitochondrion	Bel3	Poland	269 bp	2016	1–269	Pienkowska et al., 2016; Direct Submission 2016
78	KX258290	mitochondrion	Bel2	Poland	269 bp	2016	1–269	Pienkowska et al., 2016; Direct Submission 2016
79	KX258289	mitochondrion	Wiet1	Poland	269 bp	2016	1–269	Neiber & Hausdorf, 2015; Direct Submission 2016
80	KX258288	mitochondrion	KG2	Poland	269 bp	2016	1–269	Neiber & Hausdorf 2015; Direct Submission 2016
81	KM247397	mitochondrion	(haplotype 16S 8)	Poland	269 bp	2014	1–269	Pienkowska et al., 2015; Direct Submission 2014
82	KM247391	mitochondrion	(haplotype 16S 2)	Poland	269 bp	2014	1–269	Pienkowska et al., 2015; Direct Submission 2014
83	KX495378	mitochondrion	ZMH 51710-1594	Italy	843 bp	2016	1–843	Razkin et al., 2014; Direct Submission 2014
84	AY741416	mitochondrion	FG 8325	Italy	367 bp	2005	1–367	Manganelli et al., 2005; Direct Submission 2004
